# Development of Potential Multi-Target Inhibitors for Human Cholinesterases and Beta-Secretase 1: A Computational Approach

**DOI:** 10.3390/ph16121657

**Published:** 2023-11-28

**Authors:** Deyse B. Barbosa, Mayra R. do Bomfim, Tiago A. de Oliveira, Alisson M. da Silva, Alex G. Taranto, Jorddy N. Cruz, Paulo B. de Carvalho, Joaquín M. Campos, Cleydson B. R. Santos, Franco H. A. Leite

**Affiliations:** 1 Laboratório de Modelagem Molecular, Departamento de Saúde, Universidade Estadual de Feira de Santana, Feira de Santana 44036-900, BA, Brazil; deyse.brito@hotmail.com (D.B.B.); mayramosbonfim@hotmail.com (M.R.d.B.); fhpharm@gmail.com (F.H.A.L.); 2 Departamento de Informática, Gestão e Desenho, Centro Federal de Educação Tecnológica de Minas Gerais, Divinópolis 30575-180, MG, Brazil; tiago@cefetmg.br; 3 Laboratório de Bioinformática e Desenho de Fármacos, Universidade Federal de São João del-Rei, São João del-Rei 36307-352, MG, Brazil; alisson@cefetmg.br (A.M.d.S.); proftaranto@hotmail.com (A.G.T.); 4 Laboratório de Modelagem e Química Computacional, Departamento de Ciências Biológicas e de Saúde, Universidade Federal do Amapá, Macapá 68903-419, AP, Brazil; jorddynevescruz@gmail.com; 5 Feik School of Pharmacy, University of the Incarnate Word, San Antonio, TX 78209, USA; pcarvalh@uiwtx.edu; 6 Departamento de Química Orgánica Farmacéutica, Facultad de Farmacia, Campus de la Cartuja, Universidad de Granada, 18012 Granada, Spain; jmcampos@ugr.es; 7 Programa de Pós-Graduação em Biodiversidade e Biotecnologia—Rede BIONORTE, Universidade Federal do Amapá, Macapá 68903-419, AP, Brazil

**Keywords:** Alzheimer’s disease, molecular docking, molecular dynamics, pharmacophore model, triple inhibitors

## Abstract

Alzheimer’s disease causes chronic neurodegeneration and is the leading cause of dementia in the world. The causes of this disease are not fully understood but seem to involve two essential cerebral pathways: cholinergic and amyloid. The simultaneous inhibition of AChE, BuChE, and BACE-1, essential enzymes involved in those pathways, is a promising therapeutic approach to treat the symptoms and, hopefully, also halt the disease progression. This study sought to identify triple enzymatic inhibitors based on stereo-electronic requirements deduced from molecular modeling of AChE, BuChE, and BACE-1 active sites. A pharmacophore model was built, displaying four hydrophobic centers, three hydrogen bond acceptors, and one positively charged nitrogen, and used to prioritize molecules found in virtual libraries. Compounds showing adequate overlapping rates with the pharmacophore were subjected to molecular docking against the three enzymes and those with an adequate docking score (*n* = 12) were evaluated for physicochemical and toxicological parameters and commercial availability. The structure exhibiting the greatest inhibitory potential against all three enzymes was subjected to molecular dynamics simulations (100 ns) to assess the stability of the inhibitor-enzyme systems. The results of this in silico approach indicate ZINC1733 can be a potential multi-target inhibitor of AChE, BuChE, and BACE-1, and future enzymatic assays are planned to validate those results.

## 1. Introduction

Alzheimer’s disease (AD) is a disorder characterized by a progressive loss of memory and consequent loss of skills to perform common tasks [1]. AD is responsible for about 60 to 80% of non-regressive dementia cases in the world [2] but, despite its wide distribution and the resources so far dedicated to its study, its pathogenesis has not been fully elucidated. Several hypotheses have been proposed to explain AD, and the ones having so far yielded therapeutic approaches are the cholinergic and amyloid hypotheses [3,4].

The cholinergic hypothesis states that cognitive symptoms characteristic of AD are due to the loss of cholinergic neurons, with consequent reduction of acetylcholine levels in the brain. Acetylcholine levels are partially regulated by acetylcholinesterase (AChE, E.C. 3.1.1.7) and butyrylcholinesterase (BuChE, E.C. 3.1.1.8), which are responsible for the hydrolysis of excess acetylcholine released in the synaptic cleft [5]. The inhibition of those enzymes has been shown to be useful in the treatment of patients with AD [3] as it allows higher concentrations of acetylcholine to act for longer on its receptors.

The amyloid hypothesis associates AD symptoms with the formation of extracellular deposits of the β-amyloid (βA) peptide, produced by the action of proteolytic enzymes (alpha, beta, and gamma secretases) on the amyloid precursor protein (APP) [4,6]. Beta-secretase 1 (BACE-1, E.C. 3.4.23.46) starts the enzymatic process by hydrolyzing APP, producing fragments that undergo further transformation by gamma secretase to form the βA peptide. The aggregation of these peptides in extracellular clusters promotes the formation of structures that are deposited in the environment of the neuronal tissue, and contribute to progressive synaptic dysfunction, neurodegeneration, and neuronal death.

Despite representing the main form of dementia all over the world, the options for pharmacological treatment of AD patients are woefully limited, mostly based on cholinesterases and symptomatic, with low therapeutic efficacy and presenting serious adverse effects such as hepatotoxicity, hypertension, and weight loss [7,8]. A recent breakthrough was achieved with the FDA approval in 2021 of the first monoclonal antibody targeting β-amyloid aggregates [9], followed by a second one in 2023 [10]. They seem to slow down the progression of the disease and are the first examples of treatments effectively addressing the amyloid hypothesis.

Given the current scenario, the search for new drugs capable of treating symptoms, slowing down, stopping, or even reversing neurodegeneration has become one of the priorities of modern medicine. Failures in the development of anti-Alzheimer’s drugs are mainly related to the fact that much is still unknown about its causes, physiopathology, and druggable targets. Also, most approaches typically involve only one of the few known pathological pathways and disregard the multifactorial aspect of the disease [11]. The changes observed in AD patients have been shown to be interconnected, which reinforces the need for treatments capable of modulating several targets and biological systems simultaneously [12,13,14,15].

The standard therapeutic approach to reach different targets in the same disease state is to use a combination of drugs [16,17]. This approach, though proven effective throughout the years, has its own inherent risks, including the potential for adverse drug interactions and reduced patient compliance [18]. These risks are even greater in AD, as it mainly affects the elderly, who usually have associated comorbidities.

One strategy to minimize these risks is the development of drugs capable of interacting simultaneously with more than one target involved in a disease. These drugs, known as multi-target drugs [16,17,19], have shown superior results when compared to drug combination therapy in the treatment of patients with complex diseases such as cancer, with lower risk of adverse events, higher effectiveness, and lower vulnerability to biological resistance. Some multi-target drugs have been discovered by serendipity, but a more rational approach would be to use the rapidly evolving technology of molecular modeling to identify and map pharmacophores in different targets and overlap known structures, or plan the synthesis of new ones, fitting those pharmacophoric maps [17,20].

This synthetic approach, also known as molecular hybridization, creates a new chemical entity by joining two or more pharmacophore units through a ligand in order to obtain a molecule fitting the mapped pharmacophoric pockets and capable of targeting two or more enzymes or receptors [19,20]. It resembles the concept of chimeric molecules [21], but is more specific to multiple intracellular targets.

Despite the potential benefits presented by hybrid construction, this technique has limitations. Those hybrid molecules are built in a reduced chemical space, mostly limited to molecules with known activity, which were used to build/validate the pharmacophoric mapping [22]. They also tend to present structural complexity and unfavorable physicochemical properties, which make them poor drug candidates.

As previously mentioned, computer simulation techniques are becoming increasingly important in rational drug development, especially if several computational tools are used in tandem to complete and reinforce each other [23]. Computational strategies can identify essential stereo-electronic requirements for inhibition of more than one target simultaneously, using virtual screening for pharmacophore models, evaluation of interactions between molecules and the target active site and finally, molecular docking, which predicts the spatial orientation of an active compound within its binding site [24,25]. A further computational approach, molecular dynamics (MD) simulation, describes the variation in molecular behavior as a function of time, considering the system’s flexibility [26,27]. Those strategies combined can lead to more efficient structures and evaluate the potential activity of compounds not yet synthesized, based only on their chemical structure.

Employing the power of those computational tools combined, the objective of this study was to identify potential triple inhibitors against AChE, BuChE, and BACE-1 by using hierarchical virtual screening (pharmacophore models and molecular docking) and filtering by physicochemical, toxicological parameters, as well as commercial availability.

Structures evaluated in this study were retrieved from the following databases: Sigma Aldrich^®^ (St. Louis, MO, USA) (*n* = 214,446), FDA approved drug bank (*n* = 1615), Our Own Chemical Collection (OOCC) at the Federal University of São João del-Rey—UFSJ (*n* = 618), collection of thiazolidine derivatives at the Federal University of Pernambuco—UFPE (*n* = 112), and the opnMe platform from Boehringer Ingelheim (*n* = 42). 

## 2. Results

### 2.1. Pharmacophore Models Building and Validation

Known inhibitors (*n* = 50) showing inhibition higher than 30% (at 10.0 µM) and/or IC_50_ < 10.0 µM (*n* = 16) of the three targets were selected to compose the training and test sets. The compounds were clustered according to 2D structural similarity based on the Tanimoto coefficient (>0.70) [28]. From each group, molecules with the best profiles were selected for the composition of the training set (*n* = 9), and the GALAHAD^TM^ module was used to generate pharmacophore models, selecting ten of them as potential triple inhibitors (Table 1).

**Figure 1:**
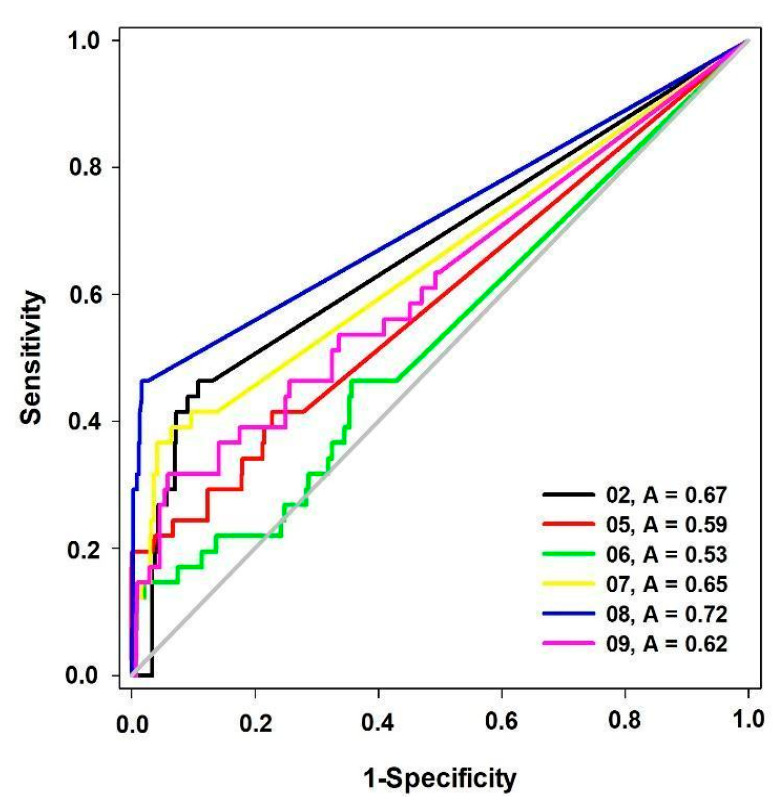
ROC curves obtained for pharmacophore models of AChE, BuChE, and BACE-1 inhibitors (A = AUC ROC).

In addition to the GALAHAD^TM^ parameters, we evaluated the pharmacophore models’ ability to differentiate active compounds (true positives) from inactive compounds (false positives = decoys), through the Receiver Operating Characteristic Curve (ROC curve) and the Area Under the ROC Curve (AUC-ROC) (Figure 1) [29].

These pharmacophore models were evaluated on their ability to identify active compounds against decoys and to assign a higher score to true positives in the initial phases of the alignment process [30,31] with the aid of early enrichment by Boltzmann-enhanced discrimination of ROC (BEDROC), presented in Table 2.

**Figure 2:**
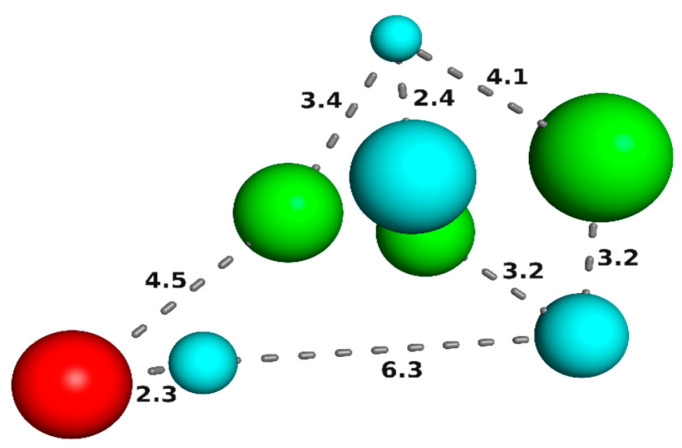
Representation of the best pharmacophore model for AChE, BuChE, and BACE-1 inhibitors (cyan spheres: hydrophobic centers; green: H-bond acceptors; red: positively charged center). The size of the spheres varies according to the tolerance radius calculated by GALAHAD^TM^. Numbers represent distances in Angstroms.

Based on the AUC and BEDROC data, model 08 (AUC = 0.72/BEDROC = 0.75) met the requirements for a reliable pharmacophore model (AUC > 0.7, BEDROC > 0.5) and was selected for the virtual screening step (Figure 2).

This pharmacophore model was previously built and validated by our group [32] and applied to a different set of databases. The results obtained here are consistent with the ones from our previous study and will expand our choices of potential triple inhibitors for synthesis, testing and further validation of our computational methods.

### 2.2. Hierarchical Virtual Screening

The structures selected were aligned to the best pharmacophore model, with 1941 showing partial overlap with QueryFit (QFIT) higher than 0.0 (2.69 < QFIT < 69.32). Seventy-one molecules meeting the criteria for mean and standard deviation (QFIT > 30.88) were selected for molecular docking assays.

A validated method [30] was used to evaluate the interactions of ligands against cholinesterases, showing the efficiency of the Auto-Dock Vina 1.1.2 program for these enzymes (RMSD_AChE_ = 1.97 Å/AUC_AChE_ = 0.88 and RMSD_BuChE_ = 1.77 Å/AUC_BuChE_ = 0.86). The tests with BACE-1 were conducted using the GOLD 5.8.1 program with the ASP scoring function (RMSD = 1.13 Å/AUC = 0.78) [33]. Compounds presenting an energy value smaller than the average of the calculated energies compared to cholinesterases (AChE < −7.95 kcal/mol and BuChE < −4.60 kcal/mol) and higher than the average of the scores calculated for BACE-1 (>37.8) (*n* = 12) were considered to have the best triple inhibition profile and selected for the prediction of toxicological and pharmacokinetic parameters.

This pharmacophore model was previously built by our group and applied to a different set of databases after thorough validation of the computational methods. The results obtained here are consistent with the ones from our previous study and will expand our choices of potential triple inhibitors for synthesis, testing and further validation of our computational methods; see Section 4.

### 2.3. Prediction of Toxicological and Physicochemical Parameters and Evaluation of Interaction Maps

The molecules prioritized by the pharmacophore model and molecular docking (*n* = 12) were evaluated for mutagenic potential through the AMES test in silico prediction [34]. Three of them showed potential mutagenicity and were discarded. The physicochemical properties of the nine remaining molecules were evaluated, and the results are presented in Table 3.

**Figure 3:**
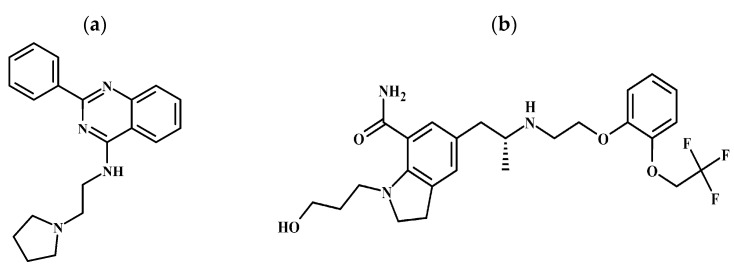
Structures selected through hierarchical virtual screening: (**a**) ZINC1733, (**b**) ZINC6063.

Three of the structures present more than one penalty regarding the parameters MW, cLogP, and number of rotatable bonds (ZINC1958, ZINC5368 and ZINC6214), as postulated by Lipinski [35] and Weber [36], and were eliminated from the study. Of the remaining six candidates, only ZINC1733 and ZINC6063 (Figure 3) are commercially available as a single enantiomer. Finding compounds commercially available is important to set the stage for future in vitro assays towards the validation of the pharmacophore model and establishment of lead structures for SAR studies. The values generated on their hierarchical virtual screenings are presented in Table 4.

**Figure 4:**
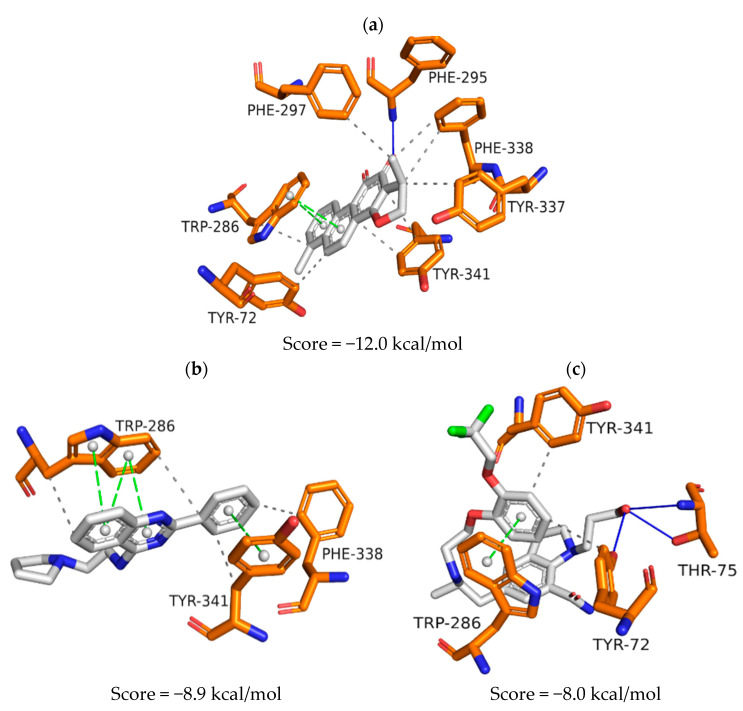
Representation of the intermolecular interactions of the crystallographic ligand dihydrotanshinone I (**a**), ZINC1733 (**b**) and ZINC6063 (**c**) at the AChE binding site.

**Figure 5:**
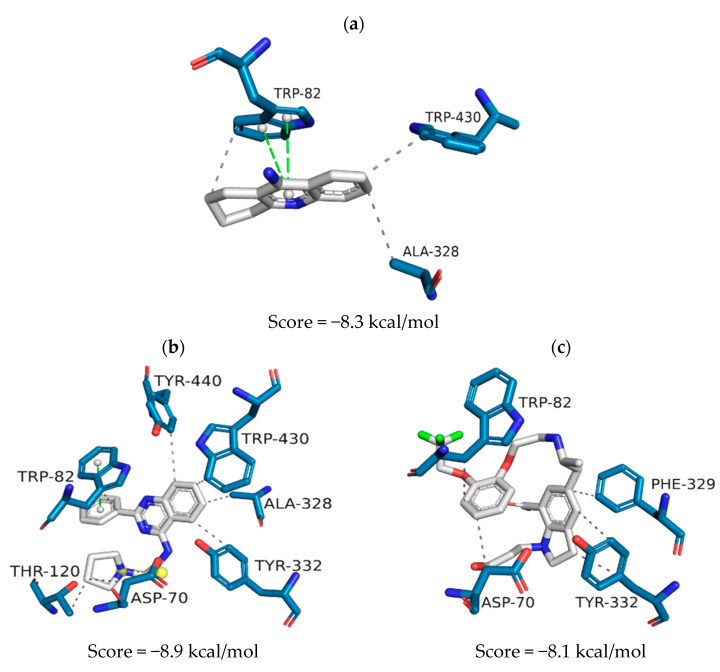
Representation of the intermolecular interactions of the crystallographic ligand tacrine (**a**), ZINC1733 (**b**) and ZINC6063 (**c**) at the BuChE binding site.

**Figure 6:**
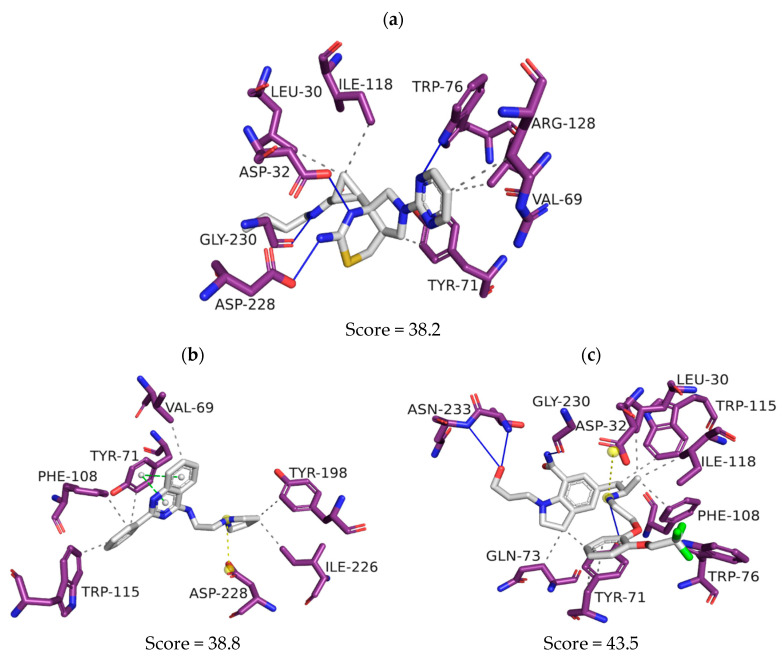
Representation of the intermolecular interactions of the crystallographic ligand 6UWP (**a**), ZINC1733 (**b**) and ZINC6063 (**c**) at the BACE-1 binding site.

Three-dimensional maps were generated to describe and evaluate intermolecular interactions established by ZINC1733 and ZINC6063 at the binding sites of the three targets (Figure 4, Figure 5 and Figure 6). For those, and all subsequent interaction maps, the following color scheme is used:

***Structures****:* white = carbons of the ligand; 

blue = nitrogen; red = oxygen; 

green = fluorine; yellow = sulfur; 

gold = carbons of amino acid residues in the AChE active site; 

blue = carbons of amino acid residues in the BuChE active site; 

purple = carbons of amino acid residues in the BACE-1 active site.

***Interactions****:* white sphere = aromatic center; yellow sphere = charged center; 

blue line = hydrogen bond; green dashed line = pi stacking interaction; 

gray dashed line = hydrophobic interaction; 

yellow dashed line = ion-ion interaction.

The AChE inhibitor and crystallographic ligand, dihydrotanshinone I (PDB ID: 4M0E), has its intermolecular interactions at the binding site presented in Figure 4a. Hydrophobic interactions are observed with residues Tyr72, Trp286, Phe297, Tyr337, Phe338, and Tyr341. Aromatic centers establish pi-stacking interactions with Trp286, and one of the oxygen atoms on the aromatic ring acts as hydrogen bond acceptor with Phe295.

The replacement of the crystallographic ligand for ZINC1733 at the AChE binding site (Figure 4b) maps hydrophobic interactions between the phenyl-quinazoline group and Trp286, Phe338, and Tyr341 and pi-stacking interactions with Trp286 and Tyr341.

Replacement of the crystallographic ligand for ZINC6063 shows hydrophobic interactions with Tyr72 and Tyr341 residues (Figure 4c) and pi-stacking between the catecholic ring and Trp286. The hydroxyl group forms hydrogen bonds with Tyr72 and Thr75.

Tacrine (PDB ID: 4BDS) is the BuChE crystallographic ligand. Its heteroaromatic ring establishes pi-stacking interactions with Trp82 (Figure 5a) and hydrophobic interactions with Trp82, Ala328 and Trp430. When tacrine is replaced by ZINC1733 in the active site of the crystallographic model, its 2-phenyl-quinazoline nucleus establishes hydrophobic interactions with Trp82, Ala328, Tyr332, Trp430 and Tyr440, as well as pi-stacking also with Trp82 (Figure 5b). At the same time, the pyrrolidine group interacts with Thr120 and Asp70, a residue with which an ion-ion bond can also be observed. ZINC6063, in turn, shows interactions of a hydrophobic nature with Trp82 andAsp70 (Figure 5c). Its indoline group, on the other hand, establishes hydrophobic interactions with Phe329 and Tyr332.

The BACE-1 crystallographic ligand (PDB ID: 6UWP) has the chemical name (1R, 2R)-2-[(4aR,7aR)-2-amino-6-(pyrim-idin-2-yl)-4a,5,6,7-tetrahydropyrrolo-[3,4-d]-[1,3] thiazin-7a-(4H)-yl]-N-{[(1R,2R)-2-methylcyclopropyl]methyl}cyclopropane-1-carboxamide (Figure 6a). Nitrogens in the thiazinamide ring form hydrogen bonds with the catalytic residues, Asp32 and Asp228, and the side chain nitrogen forms hydrogen bonds with the Gly230 residue. The pyrimidine ring forms a hydrogen bond with Trp76, and also establishes hydrophobic interactions with Val69 and Arg128, just as the pyrrole-thiazinamide nucleus establishes hydrophobic interactions with Leu30, Tyr71, and Ile118 residues.

When 6UWP is replaced by ZINC1733 in the active site of the BACE-1 crystallographic model (Figure 6b), its phenyl-quinazoline group establishes pi-stacking interactions with Tyr71 and hydrophobic interactions with Val69, Tyr71, Phe108, and Trp115. The pyrrolidine ring establishes hydrophobic interactions with Tyr198 and Ile226. The pyrrolidinyl nitrogen forms an ion-ion bond with the catalytic Asp228 residue.

ZINC6063, in turn, establishes hydrophobic interactions with Leu30, Tyr71, Gln73, Phe108, Trp115, and Ile118 (Figure 6c). It forms hydrogen bonds with Trp76, Asn233, Tyr71, and Gly230. It also forms an ion-ion bond between the tertiary amine of the ligand and the Asp32 residue.

### 2.4. Molecular Dynamics (MD)

**Figure 7:**
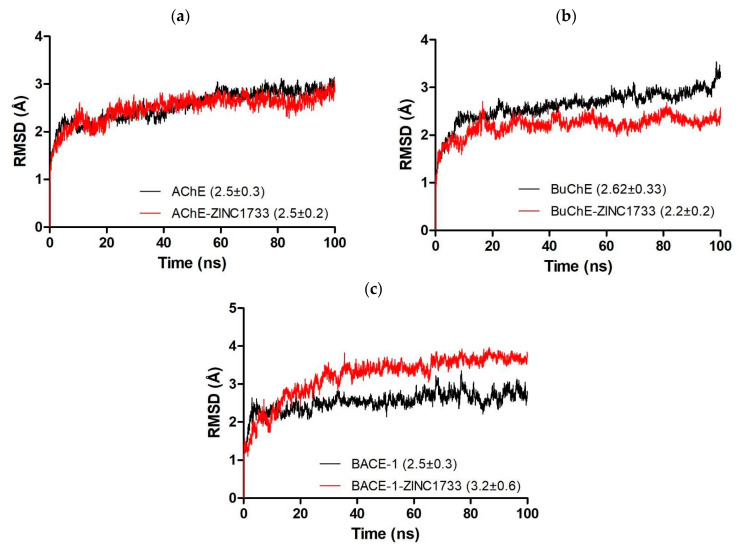
RMSD (backbone) of APO structures and AChE (**a**), BuChE (**b**), and BACE-1 (**c**) complexes with ZINC1733 during the 100 ns molecular dynamics.

MD simulations with AChE, BuChE, and BACE-1 apo and the top-ranked molecule (ZINC1733) with the three targets were performed. The systems were initially evaluated for structural stability based on the Root Mean Square Value (RMSD) along the 100 ns trajectory, and the results are shown in Figure 7.

The evaluation of the trajectories allows us to affirm that the systems achieved the equilibrium state at different times for the simulations (AChE apo: 15 ns; AChE complexed: 25 ns; BuChE apo: 20 ns; BuChE complexed: 30 ns; BACE-1 apo: 10 ns; BACE-1 complexed: 40 ns).

Those simulation steps were considered as the initial time for the productive phase for each one of the systems, until the final simulation time, 100 ns (except for the BuChE apo form, which was considered as a productive phase from 20 until 95 ns).

**Figure 8:**
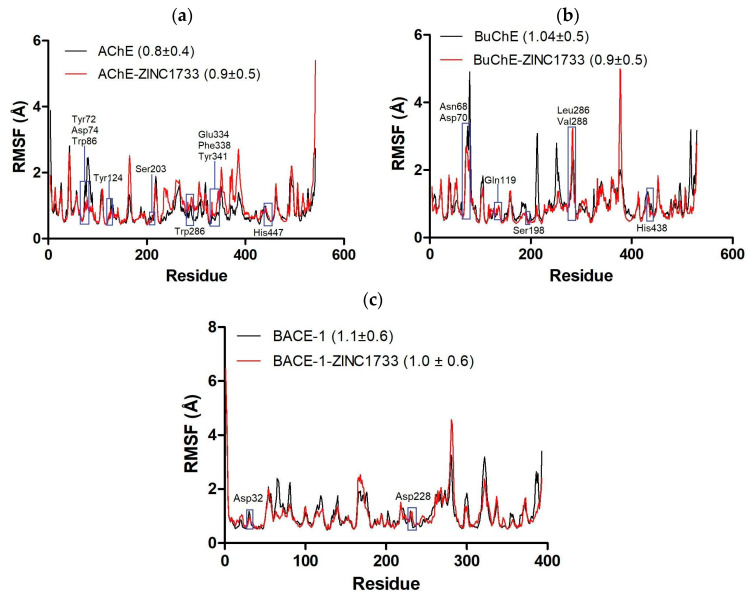
RMSF (Å) (backbone) of APO structures and complexes of AChE (**a**), BuChE (**b**), and BACE-1 (**c**) with ZINC1733 during the respective productive phase. The blue highlights correspond to the binding site regions.

Besides RMSD analysis, we evaluate the atomic fluctuations of the residues individually by calculating their root-mean-square fluctuation (RMSF). The fluctuation plots of the residues were generated for the apo form and compared with those of the respective complexes with ZINC1733, during the productive phase from each simulation (Figure 8).

Atomic fluctuation of the complex with AChE (RMSF = 0.9 ± 0.5 Å) and the APO form (RMSF = 0.8 ± 0.4 Å) in absolute terms are statistically equivalent (Figure 8a), similar to what is observed for the BuChE APO form (RMSF = 1.0 ± 0.5 Å) and complex (RMSF = 0.9 ± 0.5 Å) (Figure 8b). By graphical analysis, fluctuations are more evident for the APO form in the active binding sites of both cholinesterases, especially at the residues Tyr72, Asp74, and Trp86 from AChE and Asn68 and Asp70 from BuChE. The BACE-1 atomic fluctuations in apo form (RMSF = 1.1 ± 0.6 Å) and in the complexed form with ZINC1733 (RMSF = 1.0 ± 0.6 Å) also reveal that the system fluctuations are similar (Figure 8c) and catalytic residues show similar behavior in both systems. Similar structural behavior can be seen by PCA plot (see Supplementary Material Figure S1), in which higher fluctuation regions are preserved.

**Figure 9:**
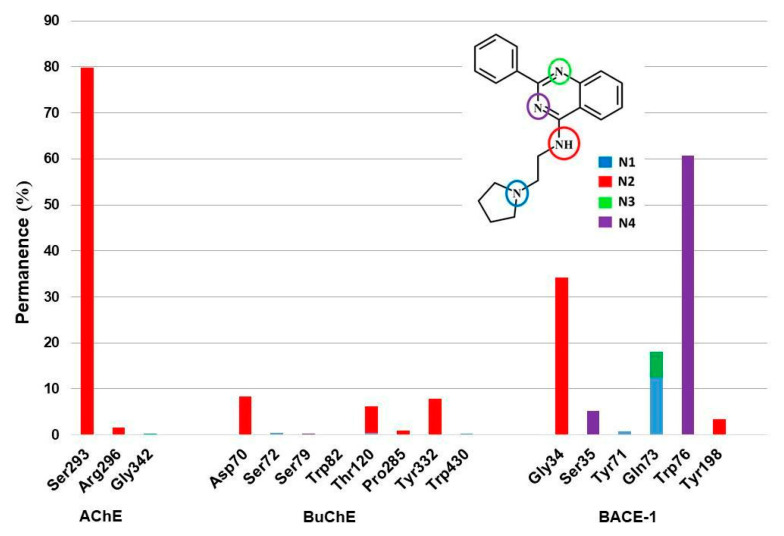
Permanence rate of hydrogen interactions (hbond) of ZINC1733 in the active site of AChE, BuChE and BACE-1 during the production phase and identification of the involved pairs.

After evaluating the system’s stability, we analyzed the interactions observed during the MD simulations. Initially, the number and permanence of hydrogen bonds established between the amino acids of AChE, BuChE, and BACE-1 active sites and ZINC1733 during the MD productive phase were evaluated (Figure 9).

At the AChE active site, only one hydrogen bond was observed for a period greater than 10%, established between the ligand nitrogen N2 and the residue Ser293 (79.88%). In BuChE, on the other hand, none of the residues was involved in hydrogen bonding with permanence greater than 10%. In the BACE-1 complex, H interactions occurring for more than 10% of the simulation time are reported with Gly34 (34.28%), Gln73 (12.48%), and Trp76 (60.73%).

**Figure 10:**
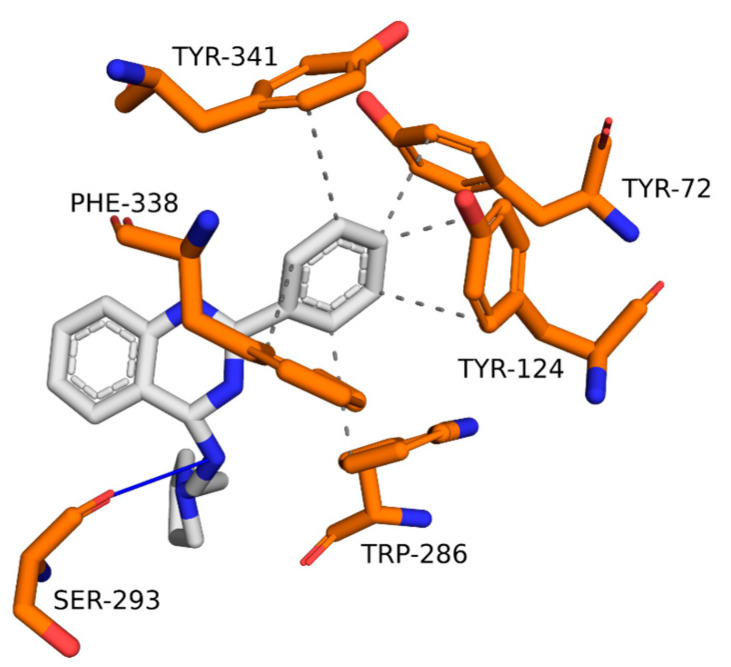
Interactions of ZINC1733 at the AChE binding site obtained from the MD simulation representative structure (69.5 ns).

**Figure 11:**
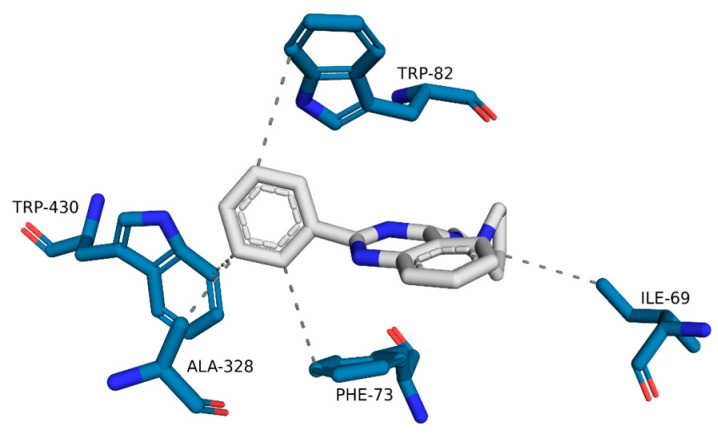
Interactions of ZINC1733 at the BuChE binding site obtained from the MD simulation representative structure (45.70 ns).

**Figure 12:**
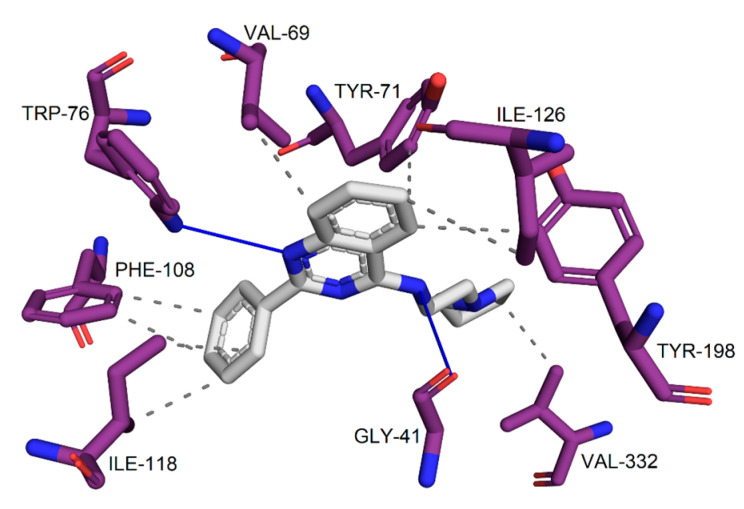
Interactions of ZINC1733 at the BACE-1 binding site obtained from the MD simulation representative structure (90.60 ns).

In addition to the information obtained from the hydrogen bonds observed during the MD simulation productive phase, we evaluated other interactions that can be established between the ligand-protein. We selected a graphical simulation representation through the analysis of different RMSD values to determine a cutoff point at the maximum RMSD value between the conformations. The evaluations indicated that, for the AChE-ligand complex, the most appropriate RMSD value for use as a cutoff point was 1.3 Å, while for the ligand complexed to BuChE and BACE-1 systems, the value was 1.2 Å. Thus, selected structures were the conformations at the time 69.65 ns for AChE-ZINC1733, 45.70 ns for BuChE-ZINC1733, and 90.60 ns for BACE-1-ZINC1733. Graphical representations are shown in Figure 10, Figure 11 and Figure 12.

The representative structure interaction map of the ZINC1733 complex in AChE (Figure 10) shows that the phenyl ring linked to the quinazoline establishes hydrophobic interactions with residues Tyr72, Tyr124, Trp286, Phe338, and Tyr341. In addition, the side chain nitrogen acts as a hydrogen bond donor to Ser293.

The representative structure of the ZINC1733 complex with BuChE (Figure 11) illustrates the occurrence of hydrophobic interactions between the phenyl ring linked to quinazoline and residues Phe73, Ala328, and Trp430. In addition, we observe hydrophobic interactions between the pyrrolidine ligand group and Ile69 residue.

The interactions map of the ZINC1733 complex representative structure in BACE-1 (Figure 12) shows one hydrogen bond established between the N atom of the quinazoline group with residue Trp76, in which the ligand acts as an acceptor. The quinazoline group is also involved in hydrophobic interactions with Val69, Tyr71, Ile126, and Tyr198, while the phenyl ring linked to this group establishes similar interactions with Phe108 and Ile118. In addition, a hydrophobic interaction is observed between the pyrrolidine ring and the residue Val332, and one hydrogen bond is established between the N side chain and Gly41, which acts as an acceptor.

Additionally, the MM/PBSA method was applied to production phases of simulations of complexes and results are presented in Table 5.

## 3. Discussion

### 3.1. Pharmacophore Model Building and Validation

The use of pharmacophore models is a widely recognized method for fast and efficient virtual screening of potential new drugs. It presents up to 30% success in recognizing bioactive molecules and can evaluate chemically diverse ligands in extensive databases [37,38]. Additionally, it does not need the full three-dimensional structure of the biological target elucidated [39], which increases its applicability.

The quality of pharmacophore models is directly associated with the original set of molecules. In our case, they need to show different chemotypes and affinity towards the three targets simultaneously. In this study, we clustered the “actives” set in groups based on the Tanimoto coefficient, as this strategy guarantees more accurate pharmacophore models [40]. Nine molecules were selected with diverse structural nuclei after the similarity assessment allowed that choice.

The GALAHAD^TM^ module was used to generate pharmacophore models and we evaluated the statistical parameters provided to select the best one (Table 1). Previous studies showed that pharmacophore models generated by energetically unfavorable conformations of the ligands are unreliable and should be excluded [41,42]. In this context, the pharmacophore models 01, 03, 04, and 10 presented an energy penalty (Energy > 100.0 kcal/mol) and were excluded. However, this parameter was not sufficient to select a single pharmacophore model. The PARETO value, which represents a normalization of the values of the quality components of the pharmacophore models (STERICS, HBOND, and MOL_QRY), was then evaluated and demonstrated that no pharmacophore model is statistically superior compared to the others [43], since all values were equal to zero. This measure was also insufficient to select the best pharmacophore model to be used in the stages of virtual screening.

Our next step was to apply enrichment metrics AUC ROC and BEDROC (Figure 1; Table 2). An ideal ROC curve grows vertically along the Y axis, symbolizing the identification of true positives (active), and proceeds horizontally to the right after reaching the maximum point, which means that decoys are not flagged by the pharmacophore model [44]. Under these ideal conditions, the value of the area under the curve would be equal to 1.0. In contrast, an area under the curve less than 0.5 corresponds to pharmacophore models with lower performance than a randomized trial [45]. To be considered predictive, a pharmacophore model must have AUC > 0.7 [46] and BEDROC value (α = 20) > 0.5 [47]. Pharmacophore model 8 (AUC = 0.72 and BEDROC = 0.75) met the requirements for a reliable pharmacophore model and, therefore, was selected for virtual screening.

Model 8 (Figure 2) has three acceptor centers for hydrogen bonding, four hydrophobic centers, and one positively charged center, characteristics described as important for activity [30,48,49,50,51,52,53]. The construction of a single pharmacophore model for the identification of potential triple inhibitors for the treatment of patients with AD consists of an innovative approach, in view of the unavailability of such models for AChE, BuChE, and BACE-1 described in the literature. Virtual screening guided by our model allows the evaluation of large libraries of compounds, which expands the chemical search space, in addition to identifying less complex molecules than classic hybrids, increasing the chances of success.

### 3.2. Hierarchical Virtual Screening

Pharmacophore model 8 was used for virtual screening of molecules present in diverse libraries, from which 71 were selected. However, this technique has limitations, such as the absence of efficient scoring metrics, based mainly on the deviation between the model and the aligned molecules, without considering compatibility with the receptor [54]. The technique also depends on databases of pre-existing conformations, allowing molecules to be neglected in screening because they do not have a specific conformation. To work around these limitations, target-based methods such as molecular docking were employed.

The combination of strategies based on ligands and target structure has been shown to be efficient in the identification of bioactive molecules, where commonly banks of molecules are subjected to filters with less computational demand, such as pharmacophore models, and then directed to computationally costlier strategies, such as molecular docking [55,56]. Thus, we subjected the 71 molecules selected through pharmacophore model virtual screening to molecular docking assays against AChE, BuChE, and BACE-1 and, with the aid of this technique, it was possible to evaluate not only the stereo-electronic characteristics necessary for triple inhibition, but the ability of the selected molecules to establish connections with the respective binding sites.

After the docking simulations, the molecules with the best score parameters (*n* = 12) were selected to predict toxicological and physicochemical parameters and evaluation of interactions established at the active site.

### 3.3. Prediction of Toxicological and Physicochemical Parameters Predictions and Evaluation of Interaction Maps

Toxicological evaluation is fundamental in the initial phase in order to optimize the drug development process. It is justified when we consider that this process is slow and expensive, taking an average of 15 years and an approximate cost of 1.3 billion dollars from the initial research phases until the market launch [57].

The main approaches to assess toxicity involve in vitro and in vivo assays, which depend on the synthesis of compounds and are not viable in large libraries [58]. For this reason, computational assays for predicting toxicity have been employed to discard those molecules with toxic potential in the initial stages. In this study, we have evaluated toxicity through in silico prediction by the Ames test, which is able to assess the possible mutagenic effects of a compound [59].

We also use in silico strategies for prediction of physicochemical parameters, as they influence the pharmacokinetics of a compound, another factor often contributing to failure in the drug development process [60]. Several computational approaches have been developed in order to predict absorption, distribution, metabolism and excretion (ADME) during the initial stages of the drug discovery [61].

The evaluation of physicochemical parameters based on Lipinski’s and Veber’s rules can predict the ability of molecules to cross biological barriers, present appropriate oral availability, and establish interactions capable of triggering a biological response [35,36]. The molecules filtered through virtual screening and Ames prediction had their physicochemical parameters evaluated and the ones with more than one penalty were discarded.

Finally, the commercial availability of the three final structures was evaluated, aiming to prioritize molecules easily accessible for preliminary biological assays of model validation. We discarded compounds produced and commercialized as a racemic mixture, as different enantiomers can promote differences in pharmacodynamics and pharmacological activity, and, at the initial stages of drug discovery, racemic mixtures are not advantageous [62].

The prioritized molecule ZINC1733 (Figure 3a) has no penalties for the physicochemical characteristics analyzed, suggesting oral bioavailability, which is fundamental in view of the chronic nature of AD and the need to establish patient compliance. From a structural point of view, it has a 2-phenyl-quinazoline nucleus that seems to be important for biological activity.

The second prioritized molecule ZINC6063 (Figure 3b) is the FDA approved drug Silodosin. It is an orally administered drug, alpha-1 adrenergic receptor antagonist, used to relieve benign prostatic hyperplasia symptoms [63]. From a structural point of view, the molecule presents an indoline group and a benzene ring that are superimposed on the aromatic centers of the triple inhibitor pharmacophore model. Regarding the physicochemical parameters, ZINC6063 presents values considered appropriate with regard to the essential characteristics of oral bioavailability, with a penalty only on the number of rotatable bonds. However, this penalty does not preclude its potential use as a drug to treat patients with Alzheimer’s, since this drug is orally administered. In addition, previous studies have shown drugs with oral bioavailability with more than 19 rotatable bonds [64].

When assessing ZINC1733 interactions at the AChE binding site (Figure 4b), it was possible to observe that the phenyl-quinazoline nucleus preserves the hydrophobic interactions profile observed at the crystallographic ligand map (with Trp286, Phe338, and Tyr341) and pi-stacking interactions (Trp286 and Tyr341). This profile is considered important for AChE inhibition, since similar interactions involving Trp286 and Phe338 are observed in AChE inhibitors with inhibitory biological activity on a nanomolar scale [65]. ZINC6063 also repeated hydrophobic and pi-stacking interactions observed with the crystallographic ligand (Tyr72, Tyr341, and Trp286) (Figure 4c). In addition, hydrogen bonds were established with Tyr72 and Thr75, which are reported to be important for AChE inhibition [66].

The BuChE crystallographic ligand (PDB ID: 4BDS) corresponds to tacrine, one of the first drugs used to treat patients with AD. The prioritized molecules repeated some of the interactions established at the BuChE active site, which reveals their potential to inhibit this target. Furthermore, the other interactions observed are described as fundamental for BuChE inhibition because the establishment of interactions with the Asp70 and Tyr332 (peripheral site) and Trp82 (anionic site) residues prevents the substrate from reaching the catalytic site of the enzyme [67]. In addition, interactions with Thr120, Phe329, Tyr332, and Tyr440 have been observed in potent BuChE inhibitors [68,69].

At the BACE-1 active site, the prioritized molecules demonstrated ability to establish interactions similar to the crystallographic ligand, although interacting only with one of the two catalytic Asp (ZINC1733 with Asp228 and ZINC6063 with Asp32). However, this fact does not imply they are not capable of inhibiting the target. Although interactions with the catalytic dyad residues (Asp32 and Asp228) in BACE-1 are remarkable for biological activity [70], interaction with at least one of the two residues is able to block the enzyme’s catalytic cycle [71]. The interactions with Tyr71 observed in the prioritized molecules, in turn, are fundamental for inhibitory activity because they promote changes in the conformation of the site, blocking it and preventing access of the substrate. Additionally, interactions established between inhibitors and residues Leu30, Val69, Phe108, Trp115, Ile118, and Ile126 are cited as important for BACE-1 inhibition [70,72,73].

In view of the discussed aspects, it is possible to state that the prioritized molecules (ZINC1733 and ZINC6063) have the stereo-electronic requirements, affinity, physicochemical requirements, and safety profile (Ames negative) appropriate to act as triple inhibitors against AChE, BuChE, and BACE-1. However, molecular docking assays do not reproduce the dynamic nature of processes occurring in a biological environment. For this reason, in order to confirm/refute the data obtained, molecular dynamics simulations were performed for the Apo forms of proteins and for the target complexes with ZINC1733, as it presents a better affinity profile towards the targets, compared to ZINC6063.

### 3.4. Molecular Dynamics (MD)

MD simulations describe in detail the variation in molecular behavior as a function of time [26]. In this study, we did simulations in order to assess the stability of the prioritized compound ZINC1733 in the active site of the enzymes AChE, BuChE, and BACE-1 as well as to evaluate the established interaction patterns.

The first metric used to evaluate the systems was RMSD, which analyzes the trajectory of the conformational changes happening in the structure protein-ligand system in relation to the unbound protein. It also identifies the moment when the system reaches equilibrium [74]. In MD simulations, it is common to experience a progressive increase in the protein RMSD value until its stabilization [26]. Figure 7 shows the systems achieving equilibrium state during the simulation time, and the RMSD values for all the systems are statistically equivalent. However, it is possible to observe a greater stability of the cholinesterases systems when in the presence of the ligand, which can be attributed to the establishment of plenty of interactions between the ligand and their binding sites. For BACE-1 systems, on the other hand, the apo system showed higher stability than when bound to the ligand, which may indicate that it promotes conformational changes.

The RMSD value, however, is not sufficient to guarantee the system stability, since it considers the entire protein structure. This data does not reveal changes occurring in the binding sites. To overcome this limitation, we evaluated the atomic fluctuations of the residues individually by calculating the RMSF during productive phases (Figure 8).

The RMSF values observed for apo and complex systems cannot be considered statistically significant. However, at the active site of cholinesterases, we observe that there is greater fluctuation for the apo systems, which suggests the occurrence of interactions between ligands and residues inducing more stability for the systems. Furthermore, previous studies have demonstrated that, on physiological conditions, BuChE changes its conformation through coordinated movements in order to allow the substrate to access the active site [75]. In the system containing the ligand, however, this movement is inhibited and the entry of the substrate is blocked. The regions where the highest fluctuations peaks are observed (Leu159-Pro166 and Gly487-Gln499 at AChE and Tyr373-Glu383 at BuChE) correspond to loop regions, which are characterized as having conformational flexibility. At the BACE-1 active site, the region containing the catalytic residues shows low fluctuation and similar comportment in both systems, which demonstrates that there were no significant changes in the conformation of the site in the simulations. The main changes in the structure of BACE-1 along the trajectory must be attributed to the loop regions located at the beginning of the chain and between Val317-Lys324, similar to what was observed at previous studies [70].

The parameters evaluated reveal that ZINC1733 forms stable complexes with AChE, BuChE, and BACE-1 and is capable of promoting useful intermolecular interactions. However, it is important to investigate the interactions responsible for the systems stabilization and that will possibly contribute to the biological response. Among these interactions, hydrogen bonding is the main interaction involved in maintaining protein structure and folding as well as molecular recognition [76]. When considering the transient aspect of hydrogen interactions, it is important to evaluate not only their occurrence, but their permanence in the simulation time.

At the AChE active site, we observed a hydrogen bond established with Ser293 during 79.88% of the simulation time. This interaction is reported to be important for the recognition of AChE inhibitors, like donepezil, since it seems to increase the ligand affinity to the referred target [77]. The BuChE active site presented a different picture. Although all the residues establishing hydrogen bonds with ZINC1733 are considered important for biologic activity [78], none of them reached more than 10% of permanence and were excluded, based on the user defined cutoff.

At the BACE-1 complex, hydrogen bonds observed occurring for more than 10% of the simulation involve residues that, although not directly involved with the catalytic mechanism, are located at the BACE-1 active site and are responsible for maintaining its conformation to guarantee substrate access to the active site [70,79]. Hydrogen interactions with Tyr71 and Gln73 have been observed in complexes with known activity BACE-1 inhibitors, where it is possible to observe the enzyme in a closed conformation blocking access to the substrate.

Despite the information obtained from the hydrogen bonding observed during the simulation of MD, it is important to note that other interactions can collaborate to the stability of the system and possible inhibition of the studied targets. To evaluate these interactions in a dynamic system, it is necessary to evaluate a graphical representation of the simulation through the selection of a representative structure of the process. The representative structures selected corresponded to the most frequent ones obtained in the groups with the highest number of conformations observed for each cutoff point.

Analysis of the interactions of AChE-ZINC1733 (Figure 10) shows the hydrophobic interactions observed in the molecular docking studies were conserved. Interactions between the phenyl ring with Tyr72 and Trp286 are described as important for the inhibitory activity [65,66], and they have been described in MD simulations involving AChE inhibitors in a complex with the protein [80]. The hydrogen bond with Ser293, although not highlighted in the interaction map from the molecular docking, has also been reported as important to increase the affinity of ligands to AChE [77]. The permanence of this connection during most of the simulation suggests it is involved in the stability of the complex.

The BuChE-ZINC1733 complex (Figure 11) reveals the establishment of hydrophobic interactions with Ala328 and Trp430, repeating what was observed in docking studies. The interaction with Trp82 (also observed in the molecular docking) was observed in MD simulations with BuChE inhibitors [81]. Additionally, we observed hydrophobic interactions between the ligand, Ile69 and Phe73, residues located at the primary entrance of the active site gorge [82]. Those interactions may promote the active site blockade of the substrate.

Although the interactions map of the ZINC1733 complex with BACE-1 (Figure 12) did not show interactions with the catalytic dyad, interactions with Val69, Tyr71, and Trp76 are reported as essential for enzymatic inhibition, since the residues are involved in attaining the enzyme transition state and in blocking the catalytic site [71,79]. This fact indicates the ligand is probably able to inhibit the enzyme. The inhibitory potential of the ligand against BACE-1 is reinforced with the interactions established with Phe108, Ile118, and Ile226, which have been previously described [83,84]. These interactions contribute to the ligand-macromolecule complex stability and can be fundamental for the stabilization of the system and triggering of the biological response.

In addition to the information obtained with MD simulations, we also calculated free energy data for the complexed systems at the production phases, as a way to evaluate the power of those biomolecular interactions. Although the most widely used method for this end in drug design is molecular docking, binding free energy measured by docking scores is not the most accurate, since it does not consider protein flexibility and solvation contributions [85]. To analyze these variables, the MM-PBSA approach has been used in association to MD simulations to compute interaction energies by combining molecular mechanics with free energy calculations based on implicit solvent models [86,87].

The binding free energy calculated indicates ZINC1733 has greater affinity for BACE-1 (−104.466 kJ/mol) than AChE (−67.980 kJ/mol) and BuChE (−80.487 kJ/mol). Additionally, it can be observed that, in all the three analyzed systems, the van der Waals interactions have the highest contribution for binding free energy when compared to electrostatic interactions. Those results illustrate that ZINC1733 binding at active sites is dominated by hydrophobic interactions, similar to what was observed in previous studies [33,88,89].

The divergences observed between representative structures and molecular docking studies can be attributed to the dynamic nature of MD simulations and confirm the fact that protein flexibility interferes with the understanding of the biological process. However, despite those divergences, the data obtained through MD simulations allows us to affirm that ZINC1733, selected by virtual screening through a pharmacophore model and molecular docking, has the necessary structural requirements to characterize it as a potential AChE, BuChE, and BACE-1 triple inhibitor.

## 4. Materials and Methods

### 4.1. Pharmacophore Model Building and Validation

#### 4.1.1. Dataset

A set of 50 triple inhibitors with biological activity data (inhibition and/or IC_50_) against AChE, BuChE, and BACE-1 was collected from the literature [90,91,92,93,94,95,96]. The 2D structure of the inhibitors was then generated in the program Marvin Sketch 19.9.0 [97] and later converted to the 3D format using the CONCORD module implemented in the SYBYL-X 2.0 program [98]. Next, the 3D structure was minimized using the Conjugate Gradient method (convergence criteria = 0.001 kcal/mol; maximum interactions = 50,000; dielectric constant = 80.0), Tripos force field [99] and partial atomic charges were assigned using the Gasteiger-Huckel method [100], as also available in the SYBYL-X 2.0 program [98].

The inhibitors (*n* = 50) were classified according to biological activity values. Those with inhibition > 30% (at 10.0 µM) and/or IC_50_ < 10.0 µM against the three enzymes (*n* = 16) were grouped based on structural similarity (Tanimoto coefficient > 0.70) [28] with the aid of the Binning Cluster tool on the ChemMine Tools server [40]. From each of the groups generated, the most active inhibitor was selected to compose the training set (*n* = 9, see Supplementary Material Figure S2), while the others were used for the validation of the generated models (see Supplementary Material Figure S3).

#### 4.1.2. Pharmacophore Model Building and Validation

The lower energy conformers of the training set were obtained through the generic algorithm implemented in the GALAHAD^TM^ module of the SYBYL-X 2.0 program [101]. For this purpose, population size and maximum number of generations for triple inhibitors were maintained at their standard values (55 and 90, respectively), as well as the advanced parameters (mutation rate = 0.4, decay rate = 1.0 and crossover rate = 1.0).

Pharmacophore models with energy values greater than 100.0 kcal/mol were discarded. The remaining were evaluated by Pareto scores, and those obtaining PARETO = 0 were evaluated for their ability to differentiate true inhibitors from false positives obtained through the DecoyFinder program [102], which provides 36 decoys for each known structure inhibitor. 

True inhibitors of the three targets not used for the pharmacophore models construction (*n* = 41) and false positives provided by DecoyFinder (*n* = 1476) were submitted to a flexible alignment to the pharmacophore models with the aid of the UNITY 3D module of the Sybyl program [103]. The alignment quality was assessed based on the overlap using the Query Fit (QFIT) value, which ranges from 0 to 100.

These values were used to construct the ROC (Receiver Operating Characteristic) curve [104] and calculation of the area under the ROC curve (AUC—Area Under the Curve) with the aid of the SigmaPlot^®^ 12.0 program [105]. In addition, the pharmacophore models were evaluated by the early enrichment rate BEDROC (Boltzmann-enhanced discrimination of ROC) with the help of the ROCKER server [106]. The pharmacophore model with a value of AUC > 0.7 and BEDROC > 0.5 (α = 20) was selected for the virtual screening.

### 4.2. Hierarchical Virtual Screening

The best pharmacophore model was used to filter molecules contained in the Sigma Aldrich^®^ catalog (http://zinc15.docking.org/catalogs/sialbb/, accessed on 15 July 2019) and in the FDA approved substance catalog (https://zinc.docking.org/substances/subsets/fda/, accessed on 1 August 2019), both on the ZINC15 platform (http://zinc.docking.org/). We also filtered molecules contained on the Our Own Chemical Collection (OOCC) platform at the Federal University of São João del-Rey (UFSJ), the library of thiazolidine derivatives from the Federal University of Pernambuco (UFPE), and the library of compounds from the opnMe platform (https://opnme.com/, accessed on 1 March 2020), granted by Boehringer Ingelheim. For this purpose, a flexible 3D alignment of the molecules contained in these banks was carried out by the UNITY^®^ 3D module, implemented in SYBYL-X 2.0. The molecules presenting a QFIT value greater than the average plus two times the standard deviation were selected for virtual screening by molecular docking.

The AChE (PDB ID: 4M0E) [107] and BuChE (PDB ID: 4BDS) [108] crystallographic structures were obtained from the Protein Data Bank and prepared using the Biopolymer module implemented in SYBYL-X 2.0, where ions and water were removed and hydrogen atoms were inserted in order to optimize hydrogen bonds. The receptors’ protonation status was adjusted to pH 7.4 on the ProKa server and the conformational search and punctuation were performed by the AutoDock Vina 1.1.2 program [109] according to previously validated parameters [30].

Molecular docking with BACE-1 (PDB ID: 6UWP) [73] followed the same procedures for the first two targets. In addition to the ions and water removal, molecules and the hydrogen atoms insertion by Biopolymer, the receptor had its protonation status evaluated by the H++ 1.0 program and pKa corrected to pH 4.5 [110]. The program selected was GOLD 5.8.1 [111], the search space was delimited by the crystallographic ligand, restricted to a 10 Å sphere, and the score was provided by the ASP function according to previously validated parameters [33].

The molecules whose energy value was lower than the average of the energies calculated for the group in the cholinesterase docking studies and higher than the average of the scores calculated for BACE-1 were evaluated about the toxicological, physicochemical, and commercial availability descriptors.

### 4.3. Prediction of the Toxicological and Physicochemical Parameters and Evaluation of Interaction Maps

The molecules selected by molecular docking virtual screening were evaluated about toxicological and physical-chemical parameters by the pkCSM server [112]. Mutagenicity information was collected by the Ames test; molecular mass (MM), calculated partition coefficient (cLogP), number of rotatable bonds, number of acceptors and donors of hydrogen bonds, and polar surface area (PSA) were evaluated too (Table 6).

Molecules showing a negative toxicity profile for the AMES test and up to one physicochemical penalty were evaluated for commercial availability. The selected molecules had the intermolecular interactions described by the Protein-Ligand Interaction Profiler—PLIP server [113]. The molecule with the best interaction profile compared to the three enzymes was selected for evaluation through molecular dynamics simulations.

### 4.4. Molecular Dynamics (MD)

The apo form of the target structure and selected compound from docking approaches were analyzed in MD simulations. The 3D coordinates of the prioritized molecule after the molecular docking studies were submitted to the ATB 3.0 server [114] for the generation of its topology. The parameters of atomic charge, bond length, torsional angles and dihedrals were obtained using the GROMOS96 54A7 force field [115]. The MD simulations were performed in the GROMACS 5.1.2 package [116], in which we adopted the GROMOS96 54A7 force field parameters, temperature 25 °C, and pressure of 1 atm.

The 3D structure of AChE was obtained from the PDB (IDAChE: 4M0E; IDBuChE: 4BDS; IDBACE-1: 6UWP), from which the crystallographic ligand, water molecules, and artifacts were removed. Non-modelled regions were built through the SWISS-MODEL server [117].

The protonation status of the acidic and basic residues of the targets were adjusted in the pdb2gmx module implemented in GROMACS 5.1.2 according to the ph 7.4 for AChE and BuChE [30] and ph 4.5 for BACE-1 [110]. The residues pKa values were evaluated on the H++ server (http://biophysics.cs.vt.edu/index.php, accessed on 1 December 2020), except for the catalytic residues of BACE-1, which were manually adjusted for the protonated (Asp32) and deprotonated (Asp228) state [118]. To solvate the systems, a dodecahedral box with water model SPC-E [119] was used, with a minimum distance of 1.4 nm from the box edges. For neutralization, in systems involving AChE (apo and complex), 7 Na^2+^ ions were added, while in systems with BuChE and BACE-1, 4 and 5 Cl^−^ ions were added, respectively.

Apo and complex systems were minimized in two steps: initially by the Steepest Descent (SD) algorithm with 10,000 cycles and, later, by the Conjugated Gradient (GC) algorithm with 1000 cycles. After the minimization steps, the equilibration step was performed (t = 1 ns) and, finally, the production dynamics (t = 100 ns) under 300 K and 1 atm. GROMACS modules (rms, rmsf and hbond functions) were utilized to analyze the stability and behavior of each system. Binding free energy of complexes were calculated by the molecular mechanics Poisson-Boltzmann Surface Area method (MM/PBSA) (g_mmpbsa tool).

## 5. Conclusions

Hierarchical virtual screening by the pharmacophore model and molecular docking, associated with molecular dynamics simulations, identified a candidate triple inhibitor against AChE, BuChE, and BACE-1. From virtual libraries, 71 molecules had QFIT > 30.88 (mean plus twice the standard deviation), which were subjected to molecular docking studies against the three targets. The molecules with the best score on docking (*n* = 12) were evaluated for toxicological, pharmacokinetic, and availability parameters. The most promising ones were selected to assess the interaction profile from molecular docking assays.

The interactions established between the prioritized molecules (ZINC1733 and ZINC6063) and the target were evaluated, identifying important interactions observed in inhibitors of known activity against the targets. In the case of interactions with cholinesterases, the molecules were able to interact with residues fundamental to the enzyme’s catalytic activity, located in peripheral and anionic sites. With BACE-1, the molecules also established important interactions with the catalytic amino acid residues, in addition to carrying out additional interactions that reinforce their binding to the active site, evidencing their ability to inhibit the activity of the enzyme.

ZINC1733, the molecule with the best affinity profile, was submitted to MD simulations. The results confirmed the interactions identified on the first steps of virtual screening and displayed additional information highlighting the inhibitory potential of the molecule. The data suggests ZINC1733 has the potential to simultaneously inhibit AChE, BuChE, and BACE-1 and is a promising lead compound for the development of new AD therapies. In vitro assays will be conducted to validate the model and confirm biological activity, followed by the synthesis of derivatives for structure-activity relationship (SAR) studies.

## Figures and Tables

**Table 1:** GALAHAD™ internal statistical parameters for pharmacophore models of AChE, BuChE, and BACE-1 inhibitors.

Model	Energy (kcal/mol)	Pareto	Sterics	HBond	Mol_qry
01 *	1450.77	0	876.2	126.8	41.05
02	62.98	0	796.1	128.0	32.26
03 *	8.71 × 10^9^	0	813.6	134.4	39.55
04 *	154.37	0	766.5	129.7	31.13
05	17.39	0	686.0	123.4	36.62
06	60.38	0	719.8	126.2	33.27
07	27.95	0	799.4	125.9	20.92
08	26.64	0	791.3	118.6	35.18
09	17.97	0	785.6	120.7	28.20
10 *	1343.38	0	811.9	118.9	36.97

* Excluded pharmacophore models.

**Table 2:** Evaluation of the early enrichment rate of pharmacophore models for the AChE, BuChE, and BACE-1 inhibitors.

Model	BEDROC (α = 20)
02	0.24
05	0.25
06	0.17
07	0.33
08	0.75
09	0.22

**Table 3:** Physicochemical properties of the 9 molecules selected by hierarchical virtual screening.

	MW (g/mol)	cLogP	Rot. Bond	HBA	HBD	PSA (Å^2^)
ZINC6063	495.542	3.0711	13	6	3	101
ZINC1733	318.424	3.8045	5	4	1	42
ZINC1958	697.924 *	6.2240 *	13 *	10	2	139
ZINC5368	509.647 *	5.4016 *	17 *	6	2	106
ZINC6214	557.054 *	5.9325 *	11 *	7	3	113
ZINC1219	480.948	4.7006	6	7	0	85
ZINC1221	480.48	4.7006	6	7	0	85
ZINC1223	480.948	4.7006	6	7	0	85
ZINC6949	409.534	3.7990	8	7	0	65

* Penalized parameters; MW = Molecular weight; cLogP = calculated partition coefficient; Rot. Bond = Number of rotatable bonds; HBA = Hydrogen Bonding Acceptors; HBD = Hydrogen Bonding Donors; PSA = Polar Surface Area.

**Table 4:** Virtual screening parameters of ZINC1733 and ZINC6063.

	QFIT	Score_AChE_ (kcal/mol)	Score_BuChE_ (kcal/mol)	Score_BACE-1_
ZINC1733	31.95	−9.3	−8.9	38.83
ZINC6063	31.67	−8.0	−8.1	43.55

**Table 5:** Binding free energy and components calculated by g_mmpbsa tools.

System	E_vdW_ (kJ/mol)	E_elec_ (kJ/mol)	G_MM_ (kJ/mol)	G_polar_ (kJ/mol)	G_nonpolar_ (kJ/mol)	ΔG_binding_ (kJ/mol)
AChE-ZN1733	−120.938	−1.557	−122.495	66.866	3.569	−67.980
BuChE-ZN1733	−144.441	−17.825	−162.266	96.478	3.516	−80.487
BACE-ZN1733	−190.126	−28.716	−218.842	132.305	3.443	−104.466

**Table 6:** Reference values of toxicological and pharmacokinetic parameters [32,33,34].

Parameter	Reference Value
Ames test	Negative
MW	<500.0 g/mol
cLogP	<5.0
Hydrogen Donors	<5
Hydrogen Acceptors	<10
Rotatable bonds	<10
PSA	<140.0 Å^2^

## Data Availability

Data is contained within the article and supplementary material.

## Supplementary Materials

The following supporting information can be downloaded at: https://www.mdpi.com/article/10.3390/ph16121657/s1, Figure S1: RMSF analysis by means of principal components analysis (PCA) for ZN1733 in complex with AChE (A), BuChE (B), and BACE-1 (C). Figure S2: 2D Chemical structures of AChE, BuChE and BACE-1 inhibitors used to build triple pharmacophore models with their biological activity data. Figure S3: 2D Chemical structures of AChE, BuChE and BACE-1 inhibitors used to pharmacophore model validation and their biological activity data.
