# Supplementary material for: Development of Potential Multi-Target Inhibitors for Human Cholinesterases and Beta-Secretase 1: A Computational Approach
Source: Pharmaceuticals (Basel). 2023 Nov 28;16(12):1657. doi: 10.3390/ph16121657 (PMC10748024; doi:10.3390/ph16121657)
Supplement: Supplementary file 1 [file pharmaceuticals-16-01657-s001.zip › pharmaceuticals-2579258-supplementary.pdf]

Article

Development of Potential Multi-Target Inhibitors for Human Cholinesterases and Beta-Secretase 1: A Computational Approach

Deyse B. Barbosa [1], Mayra R. do Bomfim [1], Tiago A. de Oliveira [2], Alisson M. da Silva [3], Alex G. Taranto [3], Jorddy N. Cruz [4], Paulo B. de Carvalho [5], Joaquín M. Campos [6], Cleydson B. R. Santos [4,7,*] and Franco H. A. Leite [1]

1 Laboratório de Modelagem Molecular, Departamento de Saúde, Universidade Estadual de Feira de Santana, Feira de Santana 44036-900, BA, Brazil; deyse.brito@hotmail.com (D.B.B.); mayramosbonfim@hotmail.com (M.R.d.B.); fhpharm@gmail.com (F.H.A.L.)

2 Departamento de Informática, Gestão e Desenho, Centro Federal de Educação Tecnológica de Minas Gerais, Divinópolis 30575-180, MG, Brazil; tiago@cefetmg.br

3 Laboratório de Bioinformática e Desenho de Fármacos, Universidade Federal de São João del-Rei, São João del-Rei 36307-352, MG, Brazil; alisson@cefetmg.br (A.M.d.S.); proftaranto@hotmail.com (A.G.T.)

4 Laboratório de Modelagem e Química Computacional, Departamento de Ciências Biológicas e de Saúde, Universidade Federal do Amapá, Macapá 68903-419, AP, Brazil; jorddynevescruz@gmail.com

5 Feik School of Pharmacy, University of the Incarnate Word, San Antonio, TX 78209, USA; pcarvalh@uiwtx.edu

6 Departamento de Química Orgánica Farmacéutica, Facultad de Farmacia, Campus de la Cartuja, Universidad de Granada, 18012 Granada, Spain; jmcampos@ugr.es

7 Programa de Pós-Graduação em Biodiversidade e Biotecnologia—Rede BIONORTE, Universidade Federal do Amapá, Macapá 68903-419, AP, Brazil

* Correspondence: breno@unifap.br

SUPPLEMENTARY MATERIAL

Figure S1. RMSF analysis by means of principal components analysis (PCA) for ZN1733 in complex with AChE (A), BuChE (B), and BACE-1 (C). The blue highlighted area corresponds to regions with more fluctuations.

Figure S2. 2D Chemical structures of AChE, BuChE and BACE-1 inhibitors used to build triple pharmacophore models with their biological activity data [90–96]. The data was obtained from published literature and the activity units were kept as presented in the original material.

Figure S3. 2D Chemical structures of AChE, BuChE and BACE-1 inhibitors used to pharmacophore model validation and their biological activity data [90–96]. The data was obtained from published literature and the activity units were kept as presented in the original material.

Figure S1. RMSF analysis by means of principal components analysis (PCA) for ZN1733 in complex with AChE (A), BuChE (B), and BACE-1 (C). The blue highlighted area corresponds to regions with more fluctuations.

(A)

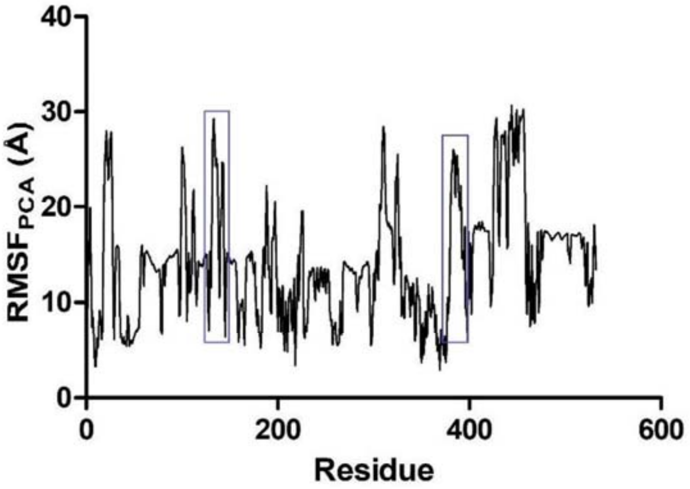

(B)

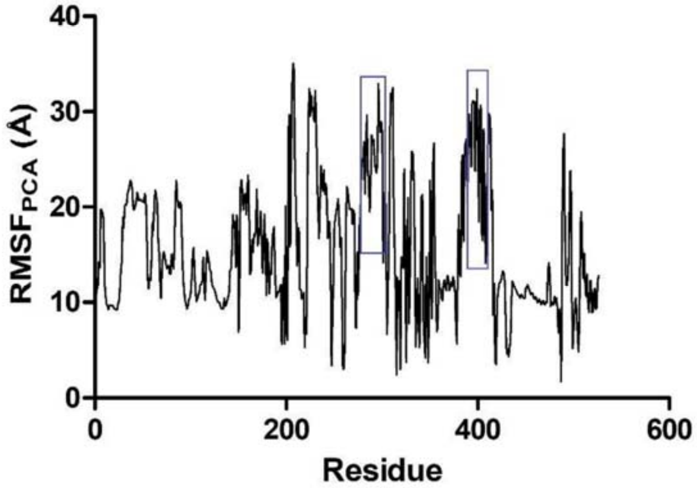

(C)

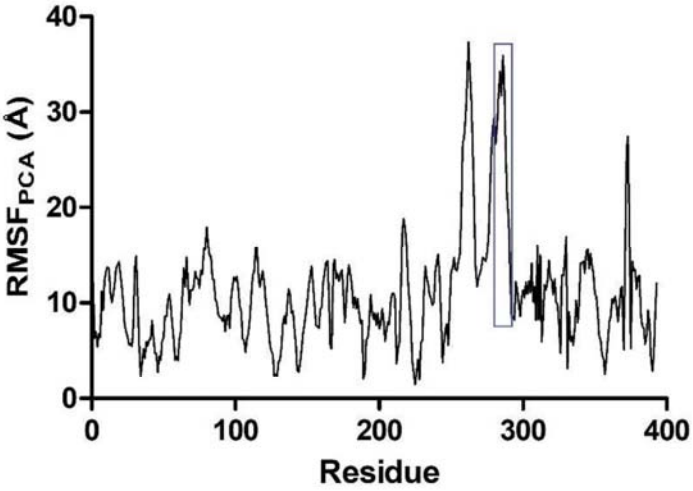

Figure S2. 2D Chemical structures of AChE, BuChE and BACE-1 inhibitors used to build triple pharmacophore models with their biological activity data [90–96]. The data was obtained from published literature and the activity units were kept as presented in the original material.

**4**	
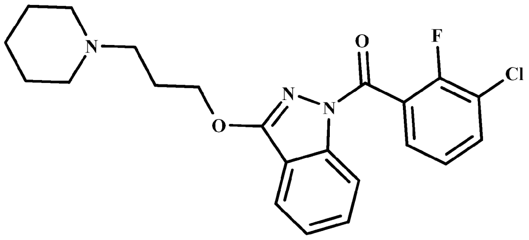	AChE % Inhibition (10 μM) = 48<br>BuChE IC_{50} = 0.80 ± 0.04 μM<br>BACE-1 % Inhibition (10 μM) = 34 ± 1
**9**	
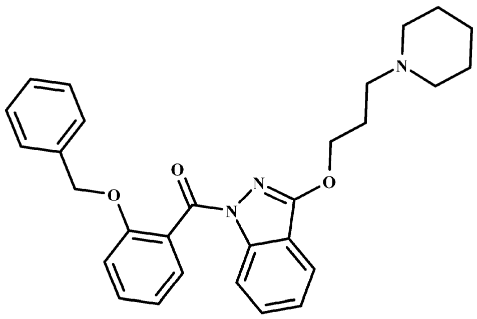	AChE%Inhibition (10 μM) = 31<br>BuChE IC_{50} = 2.5 ± 0.001 μM<br>BACE-1%Inhibition(10 μM) = 38.4 ± 1.11
**12**	
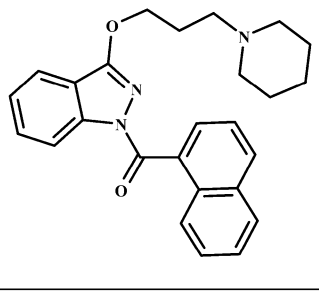	AChE%Inhibition (10 μM) = 43<br>BuChE IC_{50} = 0.007 μM<br>BACE-1%Inhibition(10 μM) = 42 ± 2
**16**	
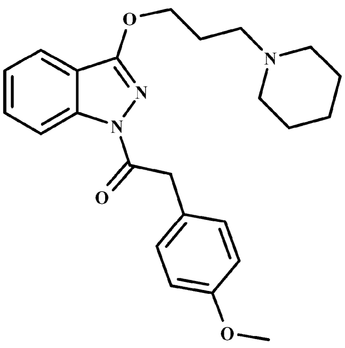	AChE%Inhibition (10 μM) = 43<br>BuChE%%Inhibition (10 μM) = 42<br>BACE-1%Inhibition (10 μM) = 60 ± 8

**18**	
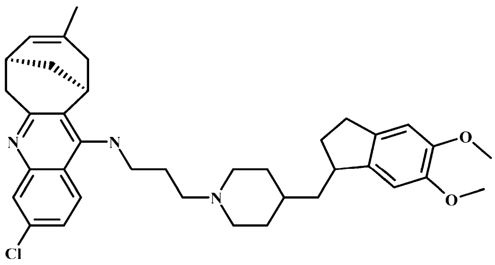	AChE IC_{50} = 0.003 μM<br>BuChE IC_{50} = 0.349 ± 0.02 μM<br>BACE-1%Inhibition(5μM) = 30,8 ± 4,1
**28**	
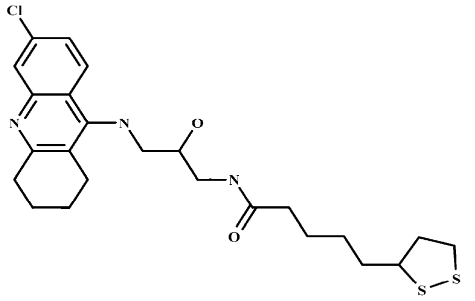	AChE IC_{50} = 0.04 μM<br>BuChE IC_{50} = 1.03 μM<br>BACE-1%Inhibition (10 μM) = 46.78 ± 3.12
**30**	
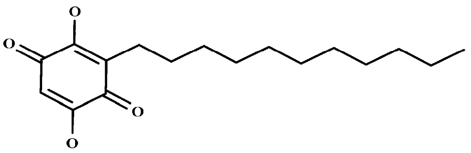	AChE%Inhibition (10 μM) = 70.15<br>BuChE%%Inhibition (10 μM) = 77.15<br>BACE-1%Inhibition (10 μM) = 77.61 ± 3.78
**40**	
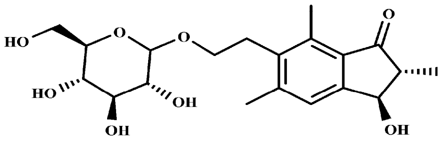	AChE IC_{50} = 5.29 ± 0.82 μM<br>BuChE IC_{50} = 3.77 ± 0.38 μM<br>BACE-1 IC_{50} = 9.74 ± 1.9 μM
**44**	
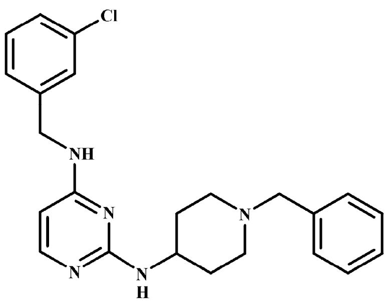	AChE IC_{50} = 7.70 ± 0.77 μM<br>BuChE IC_{50} = 2.50 ± 0.23 μM<br>BACE-1 IC_{50} = 1.70 ± 0.15 μM

Figure S3. 2D Chemical structures of AChE, BuChE and BACE-1 inhibitors used to pharmacophore model validation and their biological activity data [90–96]. The data was obtained from published literature and the activity units were kept as presented in the original material.

**1**	**2**	**3**
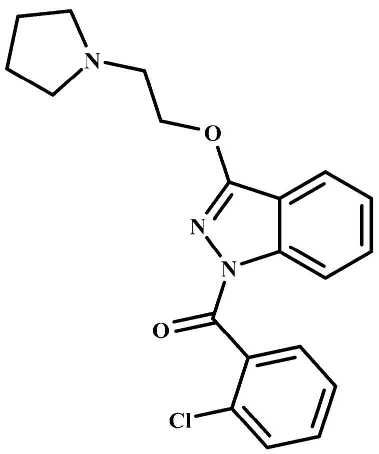	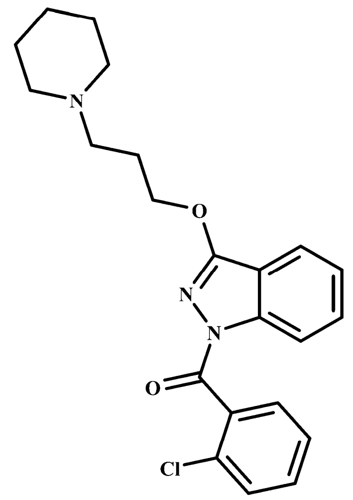	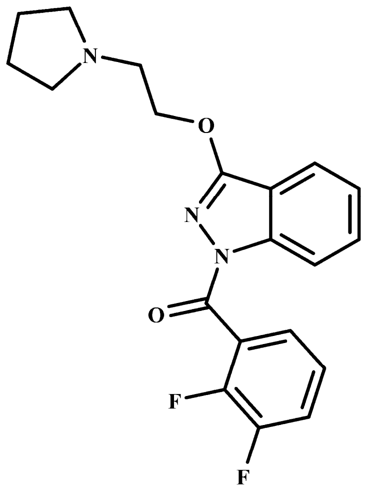
AChE IC50 = 9.7 ± 0.8 µM<br>BuChE IC50 = 1.3 ± 0.5 µM<br>BACE-1 %Inhibition (10 µM) = 9 ± 3	AChE IC50 = 9.7 ± 0.3 µM<br>BuChE IC50 = 0.07 ± 0.01 µM<br>BACE-1 %Inhibition (10 µM) = 12 ± 4	AChE IC50 = 11 ± 1 µM<br>BuChE IC50 = 11 ± 1 µM<br>BACE-1 %Inhibition (10 µM) = 14 ± 2
**5**	**6**	**7**
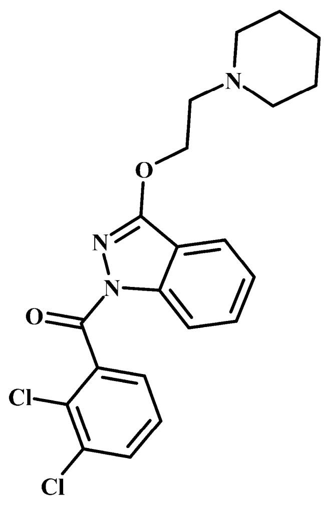	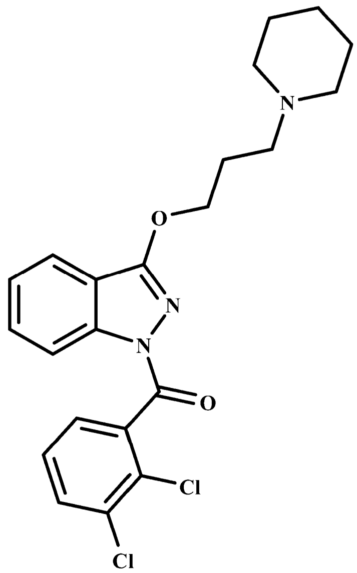	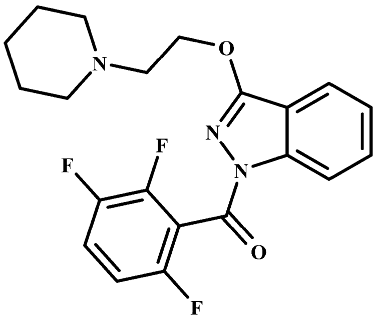
AChE IC50 = 10.5 ± 0.5 µM<br>BuChE IC50 = 0.6 ± 0.3 µM<br>BACE-1 %Inhibition (10 µM) = 18.1 ± 0.4	AChE IC50 = 17 ± 2 µM<br>BuChE IC50 = 0.080 ± 0.003 µM<br>BACE-1 %Inhibition (10 µM) = 45.1 ± 0.9	AChE IC50 = 9.4 ± 0.6 µM<br>BuChE IC50 = 9.4 ± 0.2 µM<br>BACE-1 %Inhibition (10 µM) = 14.3 ± 0.9

8	10	11
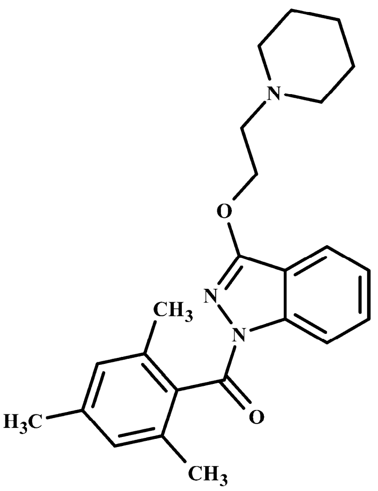	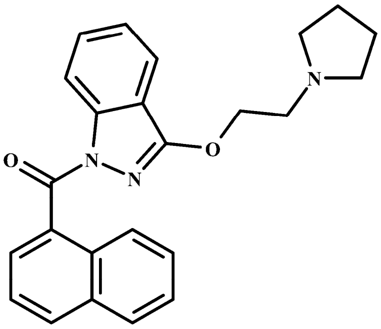	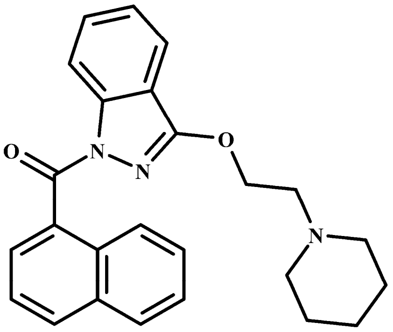
AChE IC50 = 11.6 ± 0.7 μM<br>BuChE IC50 = 0.29 ± 0.03 μM<br>BACE-1 %Inhibition (10 μM) = 12 ± 2	AChE %Inhibition (10 μM) = 35<br>BuChE IC50 = 0.15 ± 0.03 x 10-3 μM<br>BACE-1 %Inhibition (10 μM) = 20 ± 4	AChE %Inhibition (10 μM) = 37<br>BuChE IC50 = 0.26 ± 0.07 x 10-3 μM<br>BACE-1 %Inhibition (10 μM) = 11 ± 3
**13**	**14**	**15**
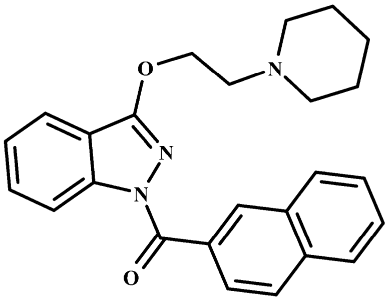	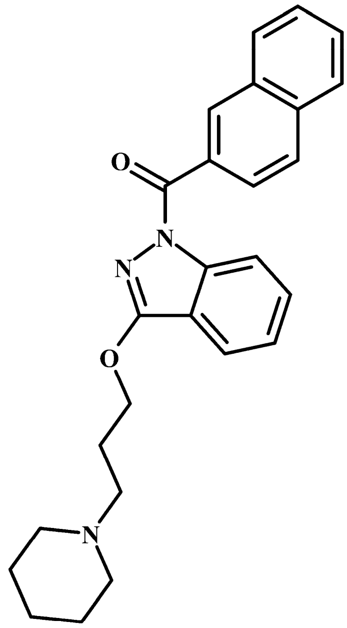	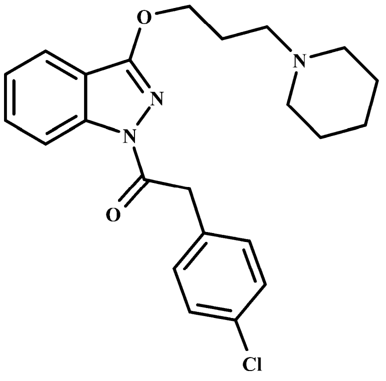
AChE %Inhibition (10 μM) = 43<br>BuChE IC50 = 4.0 ± 0.3 μM<br>BACE-1 %Inhibition (10 μM) = 50 ± 5	AChE %Inhibition (10 μM) = 33<br>BuChE IC50 = 2.1 ± 0.3 μM<br>BACE-1 %Inhibition (10 μM) = 38 ± 3	AChE %Inhibition (10 μM) = 43<br>BuChE %Inhibition (10 μM) = 46<br>BACE-1 %Inhibition (10 μM) = 50 ± 2

17	19	20
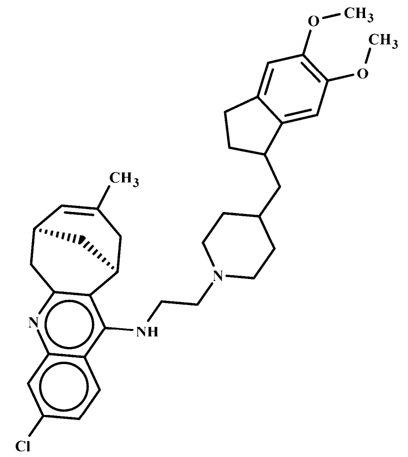	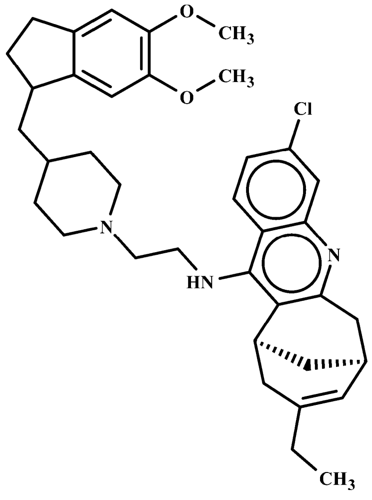	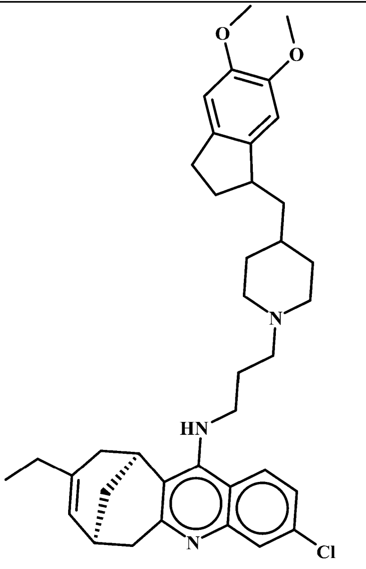
hAChE IC50 = 0.013 µM<br>BuChE IC50 = 0.303 ± 0.012 µM<br>BACE-1 %Inhibition (10 µM) = 24.6 ± 3	AChE IC50 = 0.029 ± 0.002 µM<br>BuChE IC50 = 0.419 ± 0.017 µM<br>BACE-1 %Inhibition (5 µM) = 12.5 ± 0.7	AChE IC50 = 0.0004 µM<br>BuChE IC50 = 0.194 ± 0.009 µM<br>BACE-1 %Inhibition (5 µM) = 14.9 ± 3.9
**21**	**22**	**23**
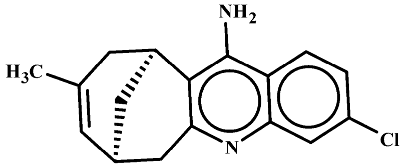	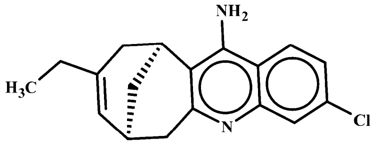	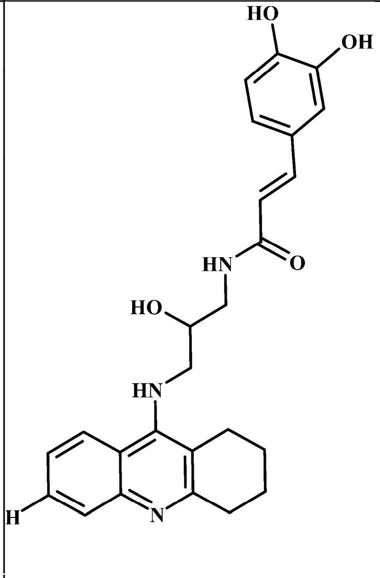
AChE IC50 = 0.004 µM<br>BuChE IC50 = 0.247 ± 0.018 µM<br>BACE-1 %Inhibition (5 µM) = 14 ± 0.1	AChE IC50 = 0.0003 µM<br>BuChE IC50 = 0.159 ± 0.01 µM<br>BACE-1 %Inhibition (5 µM) = 21.8 ± 7.2	AChE IC50 = 0.70 µM<br>BuChE IC50 = 1.01 µM<br>BACE-1 %Inhibition (10 µM) = 10.51 ± 2.55

24	25	26
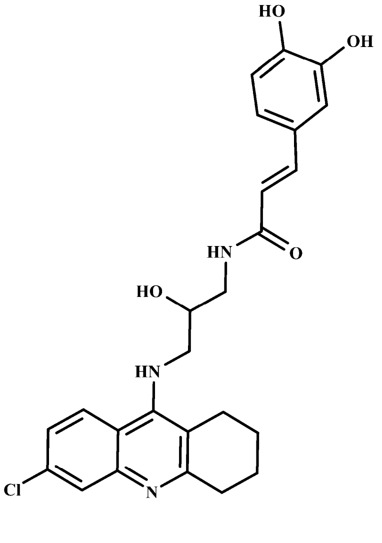	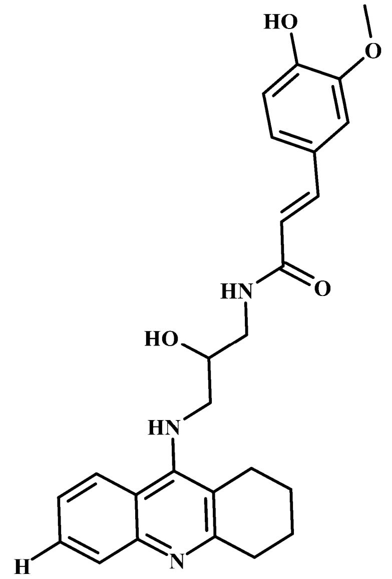	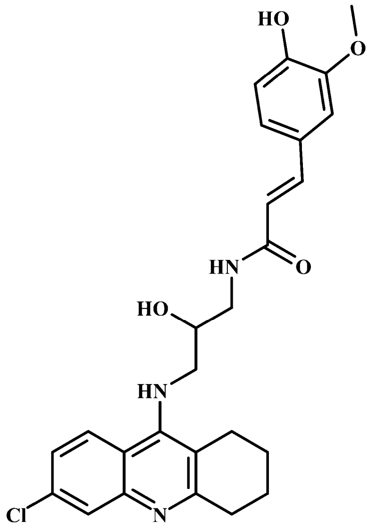
AChE IC50 = 0.15 μM<br>BuChE IC50 = 0.36 μM<br>BACE-1 %Inhibition (10 μM) = 13.07 ± 5.81	AChE IC50 = 0.03 μM<br>BuChE IC50 = 0.31 μM<br>BACE-1 %Inhibition (10 μM) = 14.97 ± 1.25	AChE IC50 = 0.02 μM<br>BuChE IC50 = 0.71 μM<br>BACE-1 %Inhibition (10 μM) = 2.57 ± 7.29
**27**	**29**	**31**
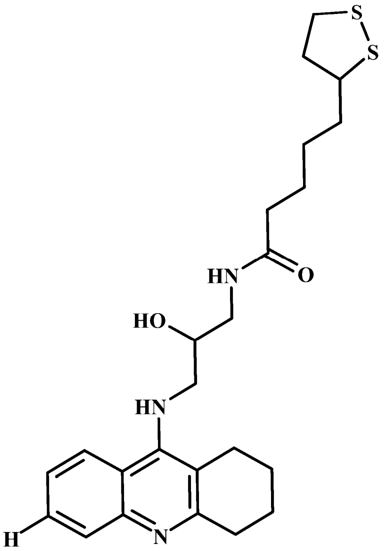	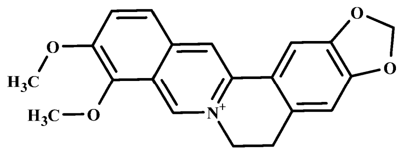	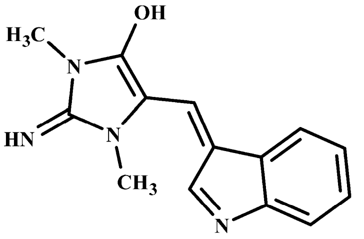
AChE IC50 = 0.10 μM<br>BuChE IC50 = 1.18 μM<br>BACE-1 %Inhibition (10 μM) = 24.77 ± 5.46	AChE %Inhibition (10 μM) = 92.05 ± 0.51<br>BuChE %Inhibition (10 μM) = 21.92 ± 1.82<br>BACE-1 %Inhibition (10 μM) = 43.42 ± 2.08	AChE %Inhibition (10 μM) = 35.62 ± 2.05<br>BuChE %Inhibition (10 μM) = 10.55 ± 0.39<br>BACE-1 %Inhibition (10 μM) = 20.32 ± 4.05

32	33	35
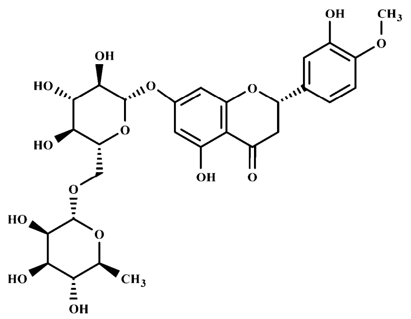<br>AChE IC50 = 22.80 ± 2.78 µM<br>BuChE IC50 = 48.09 ± 0.74 µM<br>BACE-1 IC50 = 16.99 ± 1.25 µM	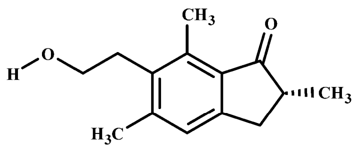<br>AChE IC50 = 16.2 ± 1.0 µM<br>BuChE IC50 = 48.1 ± 0.59 µM<br>BACE-1 IC50 = 29.6 ± 3.5 µM	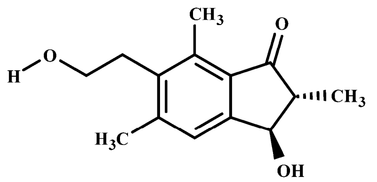<br>AChE IC50 = 17.8 ± 0.62 µM<br>BuChE IC50 = 55.9 ± 5.6 µM<br>BACE-1 IC50 = 67.1 ± 7.7 µM
**36**	**37**	**38**
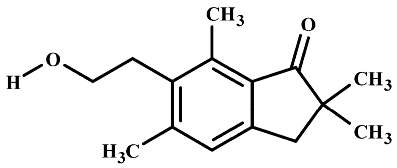<br>AChE IC50 = 46.5 ± 3.4 µM<br>BuChE IC50 = 80.1 ± 6.8 µM<br>BACE-1 IC50 = 80.0 ± 5.9 µM	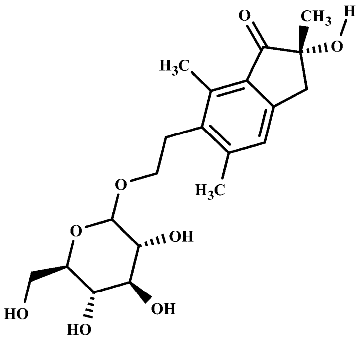<br>AChE IC50 = 110.0 ± 3.0 µM<br>BuChE IC50 = 19.4 ± 0.22 µM<br>BACE-1 IC50 = 84.6 ± 6.0 µM	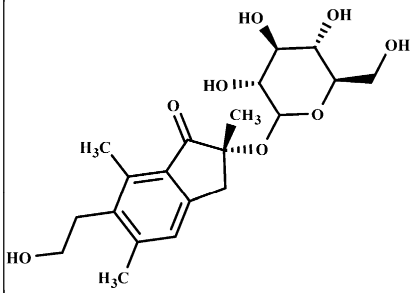<br>AChE IC50 = 39.3 ± 1.9 µM<br>BuChE IC50 = 119.0 ± 2.5 µM<br>BACE-1 IC50 = 94.4 ± 4.5 µM
**39**	**41**	**42**
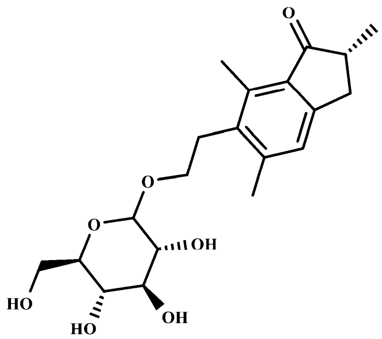<br>AChE IC50 = 2.55 ± 0.23 µM<br>BuChE IC50 = 62.0 ± 0.71 µM<br>BACE-1 IC50 = 18.0 ± 2.8 µM	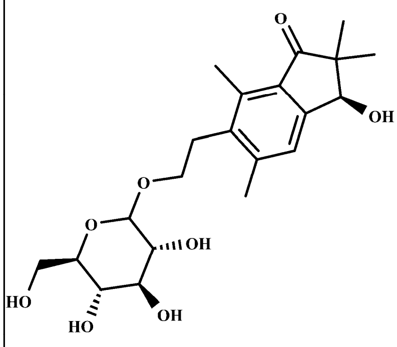<br>AChE IC50 = 27.4 ± 1.2 µM<br>BuChE IC50 = 19.3 ± 0.17 µM<br>BACE-1 IC50 = 10.7 ± 1.5 µM	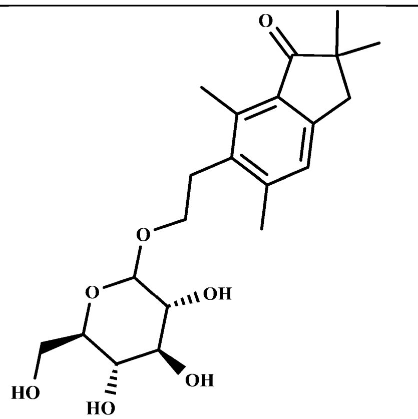<br>AChE IC50 = 24.1 ± 1.1 µM<br>BuChE IC50 = 5.31 ± 0.19 µM<br>BACE-1 IC50 = 53.3 ± 1.2 µM

43	45	46
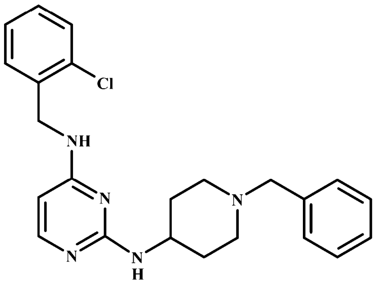	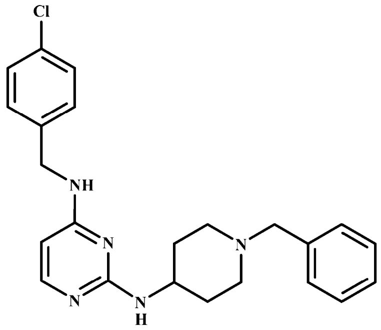	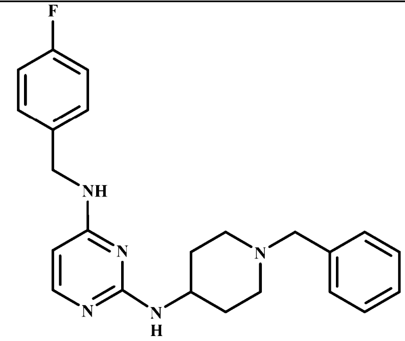
AChE IC50 = 7.0 ± 0.77 µM<br>BuChE IC50 = 2.40 ± 0.24 µM<br>BACE-1 IC50 = 1.70 ± 0.15 µM	AChE IC50 = 8.80 ± 0.88 µM<br>BuChE IC50 = 2.80 ± 0.28 µM<br>BACE-1 IC50 = 3.20 ± 0.30 µM	AChE IC50 = 7.70 ± 0.70 µM<br>BuChE IC50 = 2.20 ± 0.20 µM<br>BACE-1 IC50 = 0.70 ± 0.05 µM
**47**	**48**	**49**
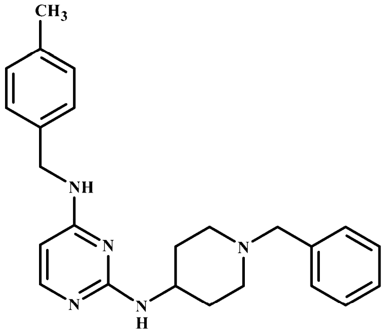	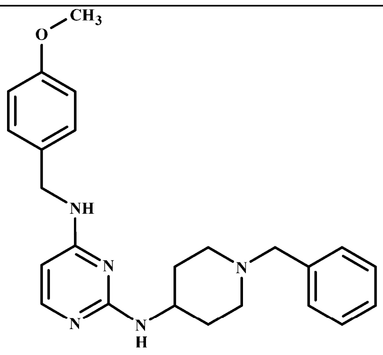	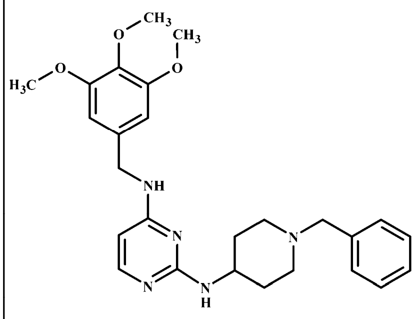
AChE IC50 = 12.90 ± 0.12 µM<br>BuChE IC50 = 2.50 ± 0.22 µM<br>BACE-1 IC50 = 11.10 ± 0.10 µM	AChE IC50 = 9.40 ± 0.90 µM<br>BuChE IC50 = 4.90 ± 0.50 µM<br>BACE-1 IC50 = 0.60 ± 0.04 µM	AChE IC50 = 10.30 ± 0.10 µM<br>BuChE IC50 = 7.70 ± 0.65 µM<br>BACE-1 IC50 = 8.90 ± 0.90 µM

50
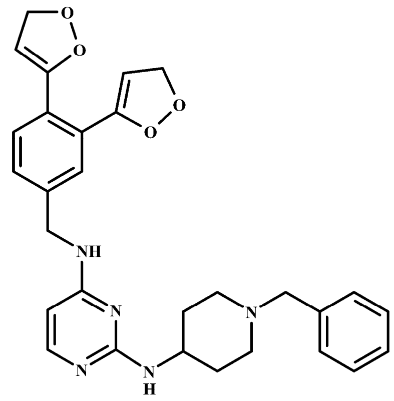
AChE IC50 = 12.60 ± 0.12 µM<br>BuChE IC50 = 3.90 ± 0.40 µM<br>BACE-1 IC50 = 1.40 ± 0.13 µM
